# The periplasmic chaperone Skp prevents misfolding of the secretory lipase A from *Pseudomonas aeruginosa*

**DOI:** 10.3389/fmolb.2022.1026724

**Published:** 2022-10-24

**Authors:** Athanasios Papadopoulos, Max Busch, Jens Reiners, Eymen Hachani, Miriam Baeumers, Julia Berger, Lutz Schmitt, Karl-Erich Jaeger, Filip Kovacic, Sander H. J. Smits, Alexej Kedrov

**Affiliations:** ^1^ Synthetic Membrane Systems, Institute of Biochemistry, Heinrich Heine University Düsseldorf, Düsseldorf, Germany; ^2^ Center for Structural Studies, Heinrich Heine University Düsseldorf, Düsseldorf, Germany; ^3^ Institute of Biochemistry, Heinrich Heine University Düsseldorf, Düsseldorf, Germany; ^4^ Center for Advanced Imaging, Heinrich Heine University Düsseldorf, Düsseldorf, Germany; ^5^ Institute of Molecular Enzyme Technology, Jülich Research Center, Jülich, Germany

**Keywords:** protein secretion, folding, aggregation, LipA, SurA, FkpA, PpiD, YfgM

## Abstract

*Pseudomonas aeruginosa* is a wide-spread opportunistic human pathogen and a high-risk factor for immunodeficient people and patients with cystic fibrosis. The extracellular lipase A belongs to the virulence factors of *P. aeruginosa*. Prior to the secretion, the lipase undergoes folding and activation by the periplasmic foldase LipH. At this stage, the enzyme is highly prone to aggregation in mild and high salt concentrations typical for the sputum of cystic fibrosis patients. Here, we demonstrate that the periplasmic chaperone Skp of *P. aeruginosa* efficiently prevents misfolding of the lipase A *in vitro. In vivo* experiments in *P. aeruginosa* show that the lipase secretion is nearly abolished in absence of the endogenous Skp. Small-angle X-ray scattering elucidates the trimeric architecture of *P. aeruginosa* Skp and identifies two primary conformations of the chaperone, a compact and a widely open. We describe two binding modes of Skp to the lipase, with affinities of 20 nM and 2 μM, which correspond to 1:1 and 1:2 stoichiometry of the lipase:Skp complex. Two Skp trimers are required to stabilize the lipase via the apolar interactions, which are not affected by elevated salt concentrations. We propose that Skp is a crucial chaperone along the lipase maturation and secretion pathway that ensures stabilization and carry-over of the client to LipH.

## Introduction

**FIGURE 1:**
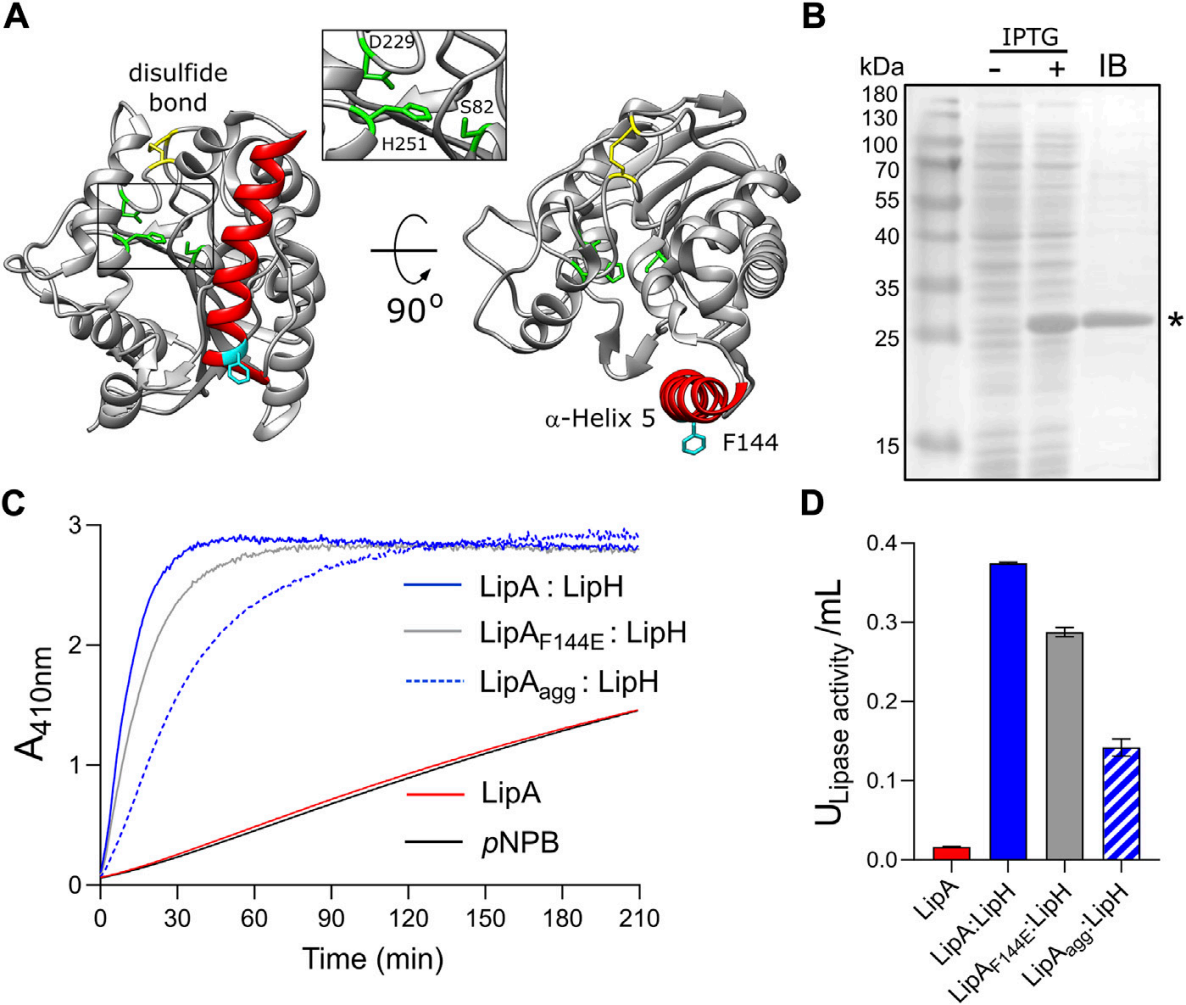
Lipase A from *Pseudomonas aeruginosa* PAO1. **(A)** Structure of the lipase LipA (PDB ID: 1EX9). The catalytic triad of Ser-82, Asp-229 and His-251 is shown in green, the disulfide bridge between Cys-183 and Cys-235 is shown in yellow. The α-helix 5 forming the lid domain is shown in red, with the residue F144 in cyan. **(B)** LipA expression and purification visualized on SDS-PAGE. Cell lysates prior to adding IPTG (“-”) and after 2 h of expression (“+”) are shown. Lane “IB”: isolated inclusion bodies. LipA band is indicated with an asterisk. **(C)** Lipase activity *in vitro* mediated by the foldase LipH. Change in absorbance at 410 nm is associated with hydrolysis of the substrate, para-nitrophenyl butyrate (*p*NPB). Once folded in presence of LipH, the wild-type LipA and the mutant LipA_F144E_ are able to hydrolyse *p*NPB (blue and grey solid traces, respectively). Pre-incubation of the wild-type lipase in the absence of LipH (LipA_agg_) leads to the partial inhibition of hydrolysis (blue dashed trace). LipA activity in the absence of LipH (red trace), and the autohydrolysis of the substrate (black trace) are indicated. **(D)** Quantification of the hydrolytic activity of LipA. The lipase activity was calculated based on the colorimetric signal after 15 min of *p*NPB hydrolysis (panel C). The assays were performed in technical triplicates, the mean values and the standard deviations (SD) are shown.

The Gram-negative bacterium *Pseudomonas aeruginosa* is a widespread opportunistic human pathogen of the highest biomedical importance, as indicated by the World Health Organization (Sadikot et al., 2005; Driscoll et al., 2007). The pathogenic potential of *P. aeruginosa* is associated with multiple secreted virulence factors, i.e. the extracellular enzymes, such as exotoxins, lipases and elastases, which facilitate the bacterial infection and adaptation pathways (Strateva & Mitov, 2011). The lipase A (LipA; Figure 1A) belongs to the most ubiquitously secreted extracellular enzymes (Lazdunski et al., 1990; Jaeger et al., 1994; Nardini et al., 2000). Secreted LipA is able to hydrolyse long- and short-chain triacylglycerols and, in cooperation with the phospholipase C, it facilitates the release of inflammatory mediators from the host cells (König et al., 1996). LipA accumulates in the biofilm matrix of *P. aeruginosa* on infected tissues, where it interacts with the bacterial exopolysaccharide alginate (Tielen et al., 2013). Although the physiological role of this interaction is not yet clarified, the biofilm assembly contributes to the bacterial growth, differentiation, and communication within the infection cycle, so the abundant LipA is seen as an element of bacterial pathogenicity (Tielen et al., 2013).

Similar to other secretory proteins, LipA is synthesised as a precursor with an N-terminal signal peptide, a hydrophobic stretch of 26 amino acids, that targets the unfolded lipase to the general SecA:SecYEG translocation pathway (Tsirigotaki et al., 2017). After the accomplished translocation across the cytoplasmic membrane, the signal peptide is cleaved off to release LipA into the periplasm for folding and maturation, followed by export via the type II secretion system (T2SS) (Douzi et al., 2011). LipA spontaneously folds into a compact intermediate state that manifests secondary and tertiary structure, however it lacks enzymatic activity (El Khattabi et al., 2000; Pauwels et al., 2012). To achieve the functional conformation prior to T2SS-mediated export, LipA of *P. aeruginosa* requires interaction with a specific foldase LipH encoded in the same operon as LipA (Hobson et al., 1993; Rosenau et al., 2004). LipH is a membrane-anchored protein with its C-terminal chaperone domain protruding into the periplasm. The available crystal structure of the homologous lipase:foldase complex from *Burkholderia glumae* (Pauwels et al., 2006) and extensive biochemical and bioinformatics analysis (El Khattabi et al., 2000; Pauwels et al., 2012; Verma et al., 2020; Viegas et al., 2020) suggest that LipH-bound LipA acquires a so-called “open state,” where its α-helix 5 is displaced as a “lid” aside to provide access to the catalytic site. Thus, LipH has been described as a steric chaperone that ensures correct positioning of the LipA lid domain, being a distinct feature of the lipase maturation pathway.

In the absence of the chaperone LipH, the non-activated LipA is prone to aggregation in the crowded bacterial periplasm, which is followed by proteolytic degradation or accumulation in inclusion bodies (Frenken et al., 1993). Alike, aggregation and proteolysis challenge the biogenesis of ubiquitous outer membrane proteins (OMPs), which, once translocated from the cytoplasm, should cross the periplasm prior to the integration into the outer membrane (Rollauer et al., 2015; De Geyter et al., 2016). To facilitate their targeting and disaggregation, a range of ATP-independent chaperones is present in the periplasm at micromolar concentrations (Masuda et al., 2009; Tsirigotaki et al., 2017). Widely conserved proteins SurA, Skp, and FkpA are the best-studied chaperones, which serve as holdases and escort client proteins to the outer membrane, while preventing their premature folding or aggregation. Studies using the model Gram-negative bacterium *Escherichia coli* have shown that the concentration of these chaperones in the periplasm may reach tens and hundreds of µM (Schmidt et al., 2016; Tsirigotaki et al., 2017). The soluble chaperones are further complemented by less-abundant PpiD and YfgM proteins anchored at the inner membrane and associated with the SecYEG translocon (Götzke et al., 2014; Sachelaru et al., 2014). Next to OMPs, the periplasmic chaperones are also involved in biogenesis of several secretory proteins (Purdy et al., 2007; Jarchow et al., 2008; Narayanan et al., 2011; Entzminger et al., 2012), but the mechanism of these interactions has been barely investigated.

Here, we demonstrate that folding of LipA is challenged by the high aggregation propensity of its lid domain, while a mutation within this region as well as interactions with the foldase LipH greatly stabilize the protein. When examining interactions of LipA with the general periplasmic chaperones of *P. aeruginosa*, we discover a potent anti-aggregation effect of the chaperone Skp, while LipA:Skp interactions do not prevent LipH-mediated activation of the lipase. Notably, the lipase secretion in *P. aeruginosa* is nearly abolished when the *skp* gene is knocked-out. The structural analysis of Skp reveals a characteristic jellyfish-shaped trimeric assembly and suggests extensive conformational dynamics. The hydrophobic central cavity within the Skp trimer may expand to accommodate a LipA molecule, although two bound copies of Skp trimer are required to prevent LipA from aggregation. The results of *in vitro* and *in vivo* experiments suggest that Skp is a crucial chaperone in the LipA biogenesis pathway that ensures client’s maturation and secretion under challenging environmental conditions.

## Materials and methods

### Molecular cloning

The genes of *P. aeruginosa* PAO1 strain were identified using *Pseudomonas* Genome database (www.pseudomonas.com). Those included PA2862 (*lipA*), PA2863 (*lipH*), PA3262 (*fkpA*), PA3801 (*yfgM*), PA 1805 (*ppiD*), PA0594 (*surA*) and PA3647 (*skp/ompH/hlpA*). The genomic DNA from *P. aeruginosa* PAO1 served as a template for the amplification of the genes of interest via PCR using the KOD Xtreme polymerase (Novagen/Merck) and cloning primers containing the restriction sites (Eurofins Genomics). The PCR products were isolated using the NucleoSpin Gel and PCR Clean-up kit (Macherey-Nagel). Standard molecular cloning techniques were further employed to insert the genes of interest into target vectors using restriction nucleases (New England Biolabs). The amplified genes encoding the full-length periplasmic chaperones were initially cloned into the pET21a vector to contain C-terminal hexa-histidine tags. The following combinations of the restriction sites were used for cloning: *BamHI/HindIII* for *skp and surA*, *EcoRI/HindIII* for *yfgM* and *ppiD*, and *EcoRI/SalI* for *fkpA*. The positions of signal peptides and membrane-anchoring domains within the chaperones were identified using SignalP-5.0 and TMHMM services, respectively (Krogh et al., 2001; Petersen et al., 2011). For overexpression of the soluble proteins, the regions encoding the N-terminal signal peptides and the membrane anchors were removed via PCR and the plasmids were re-ligated. *E. coli* strain DH5α (Thermo Fisher Scientific) served as a recipient strain for cloning and plasmid multiplication. Plasmid DNA was isolated using the NucleoSpin.Plasmid.kit (Macherey-Nagel) and analysed by Sanger sequencing (Eurofins Genomics).

### Expression and purification of periplasmic chaperones

The soluble chaperones were heterologously expressed in *E. coli* BL21(DE3), and Skp was expressed in *E. coli* BL21(DE3)pLysS. Overnight cultures were grown at 37°C upon shaking at 180 rpm and used for inoculation of pre-warmed LB medium. Cells were grown at 37°C upon shaking at 180 rpm until reaching OD_600_ 0.6, and overexpression was induced with 0.5 mM isopropyl-β-D-thiogalactopyranoside (IPTG; Merck/Sigma-Aldrich). After 2 h of expression, the cells were harvested by centrifugation at 6,000 g for 15 min, 4°C (rotor SLC-6000, Sorvall/Thermo Fischer) and resuspended in 50 mM KCl, 1 mM DTT, 10% glycerol, 20 mM HEPES pH 7.4 supplemented with protease inhibitors (cOmplete Protease Inhibitor Cocktail, Roche). The cells were lysed by shear force (M-110P cell disruptor, Microfluidics Inc.) and the debris and membranes were removed by ultracentrifugation at 205,000 g for 1 h at 4°C (rotor 45 Ti, Beckman Coulter). The supernatants were applied to IMAC with Ni^2+^-NTA-agarose resin (Qiagen) in the presence of 5 mM imidazole. After binding and extensive wash with the resuspension buffer containing 10 mM imidazole to remove weakly and non-specifically bound proteins, the chaperones were eluted with the buffer supplemented with 300 mM imidazole. The elution fractions were concentrated and subjected to SEC using Superdex 200 Increase 10/300 GL column (Cytiva). Fractions containing the chaperones were identified by SDS-PAGE, and the protein concentrations were determined spectrophotometrically based on calculated extinction coefficients at 280 nm: SurA 30940 M^−1^ cm^−1^, FkpA 8940 M^−1^ cm^−1^, PpiD 24870 M^−1^ cm^−1^, YfgM 8940 M^−1^ cm^−1^ and Skp 5960 M^−1^ cm^−1^. Monomer protein concentrations were adjusted to 50 µM and the aliquoted proteins were flash frozen and stored at −80°C. Before the experiments, the samples were thawed, centrifuged at 100.000 g, 4°C for 1 h to remove occasional aggregates and applied for SEC to match buffering conditions required for the conducted assays.

The lipase-specific foldase LipH lacking the TMD and bearing the N-terminal hexa-histidine tag was expressed heterologously as a soluble protein in *E. coli* BL21(DE3) using plasmid pEHTHis19 (Hausmann et al., 2008). LipH was purified by IMAC and subsequent SEC in 100 mM NaCl, 200 µM TCEP, 10% glycerol and 50 mM Tris-HCl pH 8.0. Samples were flash-frozen and stored at −80°C. Directly before experiments LipH samples were thawed, centrifuged at 100.000 g, 4°C for 1 h and subject to SEC in 100 mM NaCl, 10% glycerol and 20 mM Tris-HCl pH 8.0 unless other buffers are specified.

### Size exclusion chromatography combined with multi-angle light scattering

SEC-MALS was employed to probe the oligomeric states of the isolated chaperones of *P. aeruginosa*. The purified proteins were concentrated to 2 mg/ml using centrifugal filters with either 3 kDa or 30 kDa cut-off (Amicon Ultra-0.5, Merck/Millipore) and the samples were centrifuged at 100,000 g, 4°C for 1 h to remove occasional aggregates. Subsequently, 80–200 µL were injected on Superdex 200 Increase 10/300 GL column (Cytiva) connected to miniDAWN TREOS II light scattering device and Optilab-TrEX Ri-detector (Wyatt Technology Corp.). FkpA, SurA and PpiD were analysed in 50 mM KCl, 5 mM MgCl_2_ and 20 mM HEPES-KOH pH 7.4; YfgM in 5 mM glycine, 20 mM NaCl and 5 mM Tris-HCl pH 8.5; Skp in 100 mM NaCl and 20 mM Tris-HCl pH 8.0, and LipH in 100 mM NaCl, 200 µM TCEP and 50 mM Tris-HCl pH 8.0. The data analysis was performed with ASTRA 7.3.2 software (Wyatt Technology Corp.).

### Small-angle X-ray scattering

SAXS data on Skp chaperone was collected using Xeuss 2.0 Q-Xoom system from Xenocs, equipped with a PILATUS3 R 300K detector (Dectris) and a GENIX 3D CU Ultra Low Divergence X-ray beam delivery system. The chosen sample-to-detector distance for the experiment was 0.55 m, resulting in an achievable q-range of 0.05–6 nm^−1^. The measurement was carried out at 15 °C with a protein concentration of 1.20 mg/ml. Skp samples were injected into the Low Noise Flow Cell (Xenocs) via autosampler. 24 frames were collected with an exposure time of 10 minutes per frame and the data was scaled to the absolute intensity against water.

All used programs for data processing were part of the ATSAS software package, version 3.0.3 (Manalastas-Cantos et al., 2021). Primary data reduction was performed with the program PRIMUS (Konarev et al., 2003). With the Guinier approximation, the forward scattering *I(0)* and the radius of gyration (*R*_g_) were determined. The program GNOM (Svergun, 1992) was used to estimate the maximum particle dimension (*D*_*max*_) with the pair-distribution function *p(r)*. The *ab initio* reconstitution of the protein structure by dummy residues with P3 symmetry was performed using the GASBOR program (Svergun et al., 2001). The Skp trimer model was built using the cloud-based AlphaFold 2 Multimer algorithm (Evans et al., 2021; Jumper et al., 2021) and compared against the SAXS scattering data using CRYSOL (Svergun et al., 1995). The conformation analysis was performed using Ensemble Optimisation Method (EOM) using the default parameters (10000 models in the initial ensemble, native-like models, constant subtraction allowed) (Bernadó et al., 2007; Tria et al., 2015).

### Lipase isolation

The gene PA2862 encoding for the mature LipA was cloned into pET22b plasmid via *NdeI/BamHI* restriction sites (Hausmann et al., 2008) and the protein was expressed in *E. coli* BL21(DE3). Briefly, the overnight culture with LB ampicillin (100 μg/ml) grown at 37°C upon shaking at 180 rpm was used to inoculate 100 ml LB media. Cells were grown at 37°C upon shaking at 180 rpm till reaching OD_600_ 0.6. LipA expression was induced by addition of 0.5 mM IPTG to the culture and was conducted for 2 h at 37°C. Cells were harvested by centrifugation at 4,000 g, 4°C for 15 min and resuspended in 50 mM Tris-HCl pH 7.0, 5 mM EDTA, 1 mM TCEP, supplemented with 10 μg/ml DNAse I and 50 μg/ml lysozyme (buffer IB). The suspension was incubated at 20°C for 15 min and vortexed briefly, and the cells were lysed via sonication (ultrasonic homogenizer UP100H equipped with MS7 tip). The inclusion bodies were sedimented by centrifugation at 15000 g, 4°C for 10 min and suspended in the buffer IB for washing, followed by centrifugation. After repeating the procedure twice, the pellet was washed with 50 mM Tris-HCl pH 7.0. The inclusion bodies were dissolved in 8 M urea and 20 mM Tris-HCl pH 7.25, and the insoluble material was removed via a centrifugation step (21000 g, 10 min, 4°C). The protein purity was assessed by SDS-PAGE and subsequent staining (Quick Coomassie stain, Serva). LipA concentration was determined spectrophotometrically (extinction coefficient at 280 nm, ε_280_ = 27515 M^−1^ cm^−1^). LipA was aliquoted in reaction tubes (Low Protein Binding, Sarstedt), flash-frozen and stored at -80°C.

Optionally, LipA was fluorescently labelled to increase the detection efficiency in the sedimentation assay. LipA contains two endogenous cysteines in positions 183 and 235, which were targeted for site-specific fluorescent labelling. The urea-denatured LipA was incubated in 25-fold molar excess of fluorescein-5-maleimide (FM, Thermo Fisher Scientific) for 3 h at the ambient temperature. After the incubation, LipA was precipitated with 15% TCA for at least 1 h on ice. The precipitated proteins were sedimented via centrifugation at 21000 g, 4°C for 15 min, and the supernatant was removed. The pellets were washed with 0.5 ml ice-cold acetone, and then repeatedly sedimented via centrifugation at 21000 g, 4°C for 10 min. After drying at 37°C, LipA-FM pellet was solubilised in 8 M urea and 20 mM Tris-HCl pH 7.25. To remove the remaining free dye, TCA precipitation and washing steps were repeated twice. The labelling efficiency of ∼150% was determined spectrophotometrically based on the absorbance at 280 and 495 nm and the molar extinction coefficients for LipA and FM, respectively. LipA-FM was visualised on SDS-PAGE via blue-light excitation and following Coomassie blue staining.

### *In vitro* activity of the lipase

To measure the hydrolytic activity of LipA, equimolar concentrations of the foldase LipH in TGCG buffer (5 mM Tris pH 9, 5 mM glycine, 1 mM CaCl_2_, 5% glycerol) and the urea-denatured LipA (8 M urea and 20 mM Tris-HCl pH 7.25) were mixed together at concentration 1 µM in TGCG buffer to form lipase:foldase complexes in reaction tubes (Low Protein Binding, Sarstedt). The complex formation was set for 15 min at 37°C. After the incubation, 10 µL of the sample were diluted 10-fold with the TGCG buffer in 96-well plates. For the substrate preparation, 10 mM *para*-nitrophenyl butyrate, ∼1.76 μL, (*p*NPB, Merck/Sigma) were diluted from the stock solution in 1 ml acetonitrile. Immediately before starting the measurement, the substrate solution was diluted 10-fold with 50 mM triethanolamine pH 7.4 and mixed. Subsequently, 100 µL of the substrate solution was transferred to 96-well-plates with the pre-assembled LipA:LipH complex, so each well contained 0.5 mM *p*NPB and 50 nM LipA:LipH complex. For the negative control, LipA without LipH were treated the same as all other samples. To measure the autohydrolysis of the substrate, both LipA and LipH were omitted, but the corresponding buffers were added to *p*NPB. The hydrolysis of *p*NPB to *p*-nitrophenolate and butyric acid was determined by measuring the absorbance of the liberated p-nitrophenolate at 410 nm over 3.5 h at 37°C on a plate reader (Infinite 200 pro, TECAN). Samples were shaken for 5 s prior to each measurement. The hydrolytic activity of LipA was analysed by monitoring the hydrolysis based the *p*-nitrophenolate absorbance (as previously described) and by calculating the active LipA in U/mL in the linear range of the reaction (first 15 min), with the estimated molar extinction coefficient of p-nitrophenolate at 410 nm under the applied conditions of 12500 M^−1^ cm^−1^ (Filloux & Walker, 2014). The light path length in the well was experimentally determined and was of 0.53–0.55 cm upon applied conditions.

When indicated, the experiments were performed in the presence of NaCl and calcium upon LipA:LipH assembly and during the substrate hydrolysis measurements. Prior to the experiments, LipH was transferred into 100 mM NaCl, 10% glycerol and 20 mM Tris-HCl pH 8.0 by SEC, and the composition of the buffer for LipA:LipH assembly, as well as for hydrolysis measurements were adjusted. To study the effect of the periplasmic chaperones (Skp, YfgM, FkpA, SurA, PpiD) on the hydrolytic activity, the chaperones were transferred into either the TGCG buffer or 100 mM NaCl, 10% glycerol (v/v) and 20 mM Tris-HCl pH 8.0 by SEC. LipA and LipH were added in concentrations of 1 μM to form the complex, while the periplasmic chaperones were added in 5-fold molar excess. The complex formation and hydrolysis measurements were performed as described above. The activation of LipA by the periplasmic chaperones was also determined by adding LipA to the periplasmic chaperones at 1:5 M ratio in the absence of LipH. To probe the effect of the periplasmic chaperones on LipA aggregation prior to the activation, 1 μM LipA was incubated with individual periplasmic chaperones for 15 min at 37°C. Afterwards, 1 μM LipH was added to the mixtures and incubated for 15 min at 37°C. The measurements of *p*NBP hydrolysis were performed as described above.

### Light scattering assay of LipA aggregation

For the assay, either urea, TGCG buffer or 20 mM Tris-HCl pH 8.0 supplemented with NaCl were filtered using 0.1 µm syringe filters. 68.8 µL of each buffer was loaded in 96-well plates with flat transparent bottom. The urea-denatured LipA was diluted with the TGCG buffer from 150 µM stock solution to 1.6 µM, and 31.2 µl were added to 96-well microtiter plates, so the final LipA concentration was 0.5 µM. The residual urea concentration was approx. 25 mM. The samples were incubated for 1 h at 37°C, while recording the optical density at 320 nm with intervals of 30 s (Infinite M200 PRO plate reader, Tecan Trading AG). For each condition, three technical replicates were performed.

### Sedimentation analysis of LipA aggregation

LipA aggregation at various conditions was probed by sedimentation assay. To improve the detection sensitivity and so facilitate the experiment at low lipase concentrations, site-specific fluorescent labelling of the lipase was introduced. The endogenous cysteines at positions 183 and 235 were conjugated with FM, as described above, so as little as 5 ng LipA-FM could be detected via in-gel fluorescence imaging. The urea-denatured LipA-FM was diluted with the TGCG buffer from 150 µM stock solution to 1.6 µM and kept on ice protein in reaction tubes (Low Protein Binding, Sarstedt). The chaperones were transferred to 1.5 ml polypropylene reaction tubes containing 20 mM Tris-HCl pH 8.0 and varying NaCl concentrations, to achieve the specific ionic strength indicated for each assay. The minimal ionic strength conditions were probed when using the TGCG buffer. 15.6 µL of LipA-FM were added to the tube and mixed by pipetting, so the reactions contained 0.5 µM LipA-FM and 5 µM of individual chaperones in the total volume of 50 µl. For the dimeric FkpA and trimeric Skp, the monomer concentrations were 10 and 15 μM, respectively. The LipA-specific foldase LipH was used in the equimolar ratio to the lipase (final concentration 0.5 µM). The reaction tubes were incubated at 37°C for 15 min to promote LipA aggregation and the samples were then centrifuged (21000 g, 15 min, 4°C) to sediment the aggregates. To avoid disturbing the pellets, 40 µl of each supernatant fraction were transferred to new 1.5 ml tubes. The remaining material contained the pelleted LipA and 10 µL of the supernatant fraction. Both samples were precipitated by adding 100 µl of 20% TCA and incubating for 15 min on ice. The precipitated proteins were pelleted upon centrifugation at 21000 g, 4°C for 10 min, TCA was removed, and the samples were washed, as described above. Further, 15 μl of the reducing SDS-PAGE sample buffer were used to wash the tube walls, 5 μL of the sample were loaded on SDS-PAGE and in-gel fluorescence was recorded. The signal was quantified by ImageQuant software (Cytiva). The background was subtracted using the local average algorithm. The signals of the supernatant (*I*_sol_) represent the soluble fraction of LipA and correspond to 80% of the total soluble LipA. The value was used then to calculate the actual signal intensities of the soluble (*I*_sol, corr_) and aggregated (*I*_agg, corr_) LipA, as:

$$I_{sol,\;corr}\;=\;1.25*I_{sol}$$

Iagg, corr = Iagg−0.25 ∗Iso l

where *I*_agg_ is the signal measured for the aggregated LipA and the remaining 10 µL of the supernatant. The soluble fraction of LipA was calculated as a ratio:

$$\frac{I_{sol,\;corr}}{I_{sol}-I_{agg}}$$

Each assay was carried out in triplicates.

### Amyloid-specific ThT fluorescence

Thioflavin T (ThT, Merck/Sigma-Aldrich) was dissolved to the concentration of 100 μM in the buffer TGCG, and the solution was kept on ice. Immediately prior to the experiment, ThT was diluted with the TGCG buffer to 10 μM and mixed with 0.5 μM urea-denatured LipA and 5 μM chaperones. For the dimeric FkpA and trimeric Skp, the monomer concentrations were 10 and 15 μM, respectively. The total volume was set to 65 μl, where the residual urea concentration was approx. 60 mM, and the reaction was carried out at 20°C in reaction tubes (Low Protein Binding, Sarstedt). For the control experiments, containing either the chaperones or LipA, the reaction volume was adjusted to 65 μl with an appropriate buffer. After incubation at 37°C for 15 min, each reaction was transferred into a quartz cuvette for measuring ThT fluorescence at the fluorescence spectrophotometer (Fluorolog 3, Horiba Scientific). The excitation wavelength was set at 450 nm, and the emission spectra were recorded from 467 to 520 nm. ThT fluorescence intensity at 485 nm was used to evaluate and compare LipA aggregation between samples. Each measurement was carried out in independent triplicates.

### Microscale thermophoresis

MST was used to monitor interactions of Skp from *P. aeruginosa* with FM-labelled LipA_F144E_. LipA_F144E_ was diluted to 100 nM in the TGCG buffer supplemented with 0.05% Tween 20 and kept on ice protected from light. For the MST measurement, 10 µl of 50 nM LipA were mixed with Skp ranging from 2.3 nM to 18.5 µM in a 0.5 ml reaction tube (Low Protein Binding, Sarstedt). LipA:Skp samples were incubated for 15 min at 22°C in the dark, then loaded into Premium-type capillaries and analysed in Monolith NT.115 instrument (NanoTemper Technologies, Munich, Germany). The MST power was set to 80% and the LED power in the blue channel was set to 60%. The thermophoresis was detected by the normalised fluorescence time traces for 30 s with 5 s delay and 5 s for recovery. The putative LipA aggregation at the capillary surface was controlled by recording the fluorescence intensity profiles of individual capillaries before and after the experiment (time difference approx. 1 h). The data was evaluated by NT Analysis software (NanoTemper Technologies, Munich, Germany). Each sample was analysed twice, and the measurement were performed in independent triplicates.

### Transmission electron microscopy

TEM was utilized to visualize the formation of LipA aggregates. All buffers were filtered with 0.1 μm syringe filters (Whatman Puradisc). Denatured LipA was diluted to 300–500 µM in 8 M urea and 20 mM Tris-HCl pH 7.25 and then further diluted to 3–5 μM in either 8 M urea and 20 mM Tris-HCl pH 7.25, TGCG buffer or in 150 mM NaCl, 10% glycerol and 20 mM Tris-HCl pH 8.0. Samples containing Skp or LipH were prepared in 150 mM NaCl, 10% glycerol and 20 mM Tris-HCl pH 8.0 to promote lipase aggregation and examine the anti-aggregating effect of the chaperones. LipA:LipH samples contained 5 μM LipA and 15 μM LipH (molar ratio 1:3) and LipA:Skp_3_ samples contained 3 μM LipA and 15 μM Skp_3_ (molar ratio 1:5). All samples were incubated for 15 min at 37°C and used for grid preparation on a carbon-coated copper grid. 3 μl of sample were added on top of the grid incubated for 1 min at the ambient temperature. Excess liquid was removed using filter paper. For negative staining, the grid was incubated in 2% uranyl acetate (solution in distilled water pH 4.3) for 1 min in the dark. Afterwards, excess liquid was removed. Grids were dried at the ambient temperature for at least 15 min prior TEM imaging. TEM images were acquired at the Zeiss EM902 operating at 80 keV using a slow-scan CCD-Camera (Typ 7899 inside) controlled by the imaging software ImageSP (SYSPROG, TRS) and prepared using the image processing software “Fiji” (Schindelin et al., 2012).

### Analysis of LipA secretion *in vivo*

The genomic DNA of *P. aeruginosa* PAO1 isolated with the DNeasy blood and tissue kit (QIAGEN, Germany) was used as the PCR template to amplify the *lipA-lipH* operon using Phusion® DNA polymerase (Thermo Fisher Scientific, Darmstadt, Germany). The pGUF-lipAH plasmid for gene expression in *P. aeruginosa* was constructed by ligation of the PCR product into the pGUF vector (Babic, 2022) at AflII and SmaI restriction sites. Molecular cloning was conducted in *E. coli* DH5α, and the plasmid assembly was confirmed by Sanger sequencing (Eurofins Genomics). *P. aeruginosa* PA14 wild-type and Δ*skp* strains (Klein et al., 2019) transformed with pGUF (empty vector, EV) and pGUF-lipAH were cultivated in triplicates in Erlenmeyer flasks in 10 ml autoinduction medium (Terrific Broth medium containing 0.04% lactose (w/v) and 0.2% glucose (v/v)) supplemented with 100 μg/ml tetracycline. Plates were shaken at 600 rpm, volume 1.2 ml, for 24 h at 37°C. Cells were harvested by centrifugation at 4,000 g, 4°C for 10 min and the culture supernatants were filtered through 0.22 µm pore size cellulose acetate filter to remove the remaining cells. The cell pellets and the culture supernatants were kept on ice to prevent proteolysis. The supernatant concentrations were adjusted based on absorbance at 280 nm. 40 μl of the mupernatnts were diluted to final volume of 80 μl with TGCG buffer and further used for measuring the lipolytic activity of the secreted enzymes. The measurements were conducted in technical duplicates for all biological triplicates, thus 40 μl of sample were put in 96-well microtiter plates, and 40 uL of 1 mM of *p*NPB were added for measuring the esterase activity. The hydrolytic activity of the secreted elastase LasB was determined using Abz-Ala-Gly-Leu-Ala-*p*-nitro-benzyl-amide substrate (Echelon Biosciences) (Zhu et al., 2015). Supernatants were diluted 20-fold with 100 mM Tris-HCl pH 7.5 and 20 µl of each supernatant was loaded in 96-well microtiter plates. Prior to measurement, 250 mM substrate solution in 50 mM Tris-HCl pH 7.0, 2.5 mM CaCl_2_, 1% DMSO (v/v) was added to the samples in volumes of 100 µl. The increase in fluorescence (λ_ex_ = 340 nm, λ_em_ = 415 nm) was monitored for 30 min at 37°C. As a positive control, the substrate was incubated with 0.3 units of proteinase K (Thermo Scientific, PCR grade). To measure the oxidase activity, the substrate solution (50 mM Tris-HCl pH 7.4, 0.12 mM NADH, 0.2 mM DTT) was added to the diluted supernatants in volumes of 100 µl per well prior to measurement. *E. coli* DH5α cells lysed via sonication (Sonotrode) were used as a positive control. The decrease in absorbance at 340 nm due to the enzymatic conversion of NADH to NAD^+^ was measured for 2 h at 23°C.

For immunoblotting the secreted proteins from the supernatant fractions were 5-fold concentrated by precipitation with trichloroacetic acid and suspension with SDS-PAGE sample buffer (100 mM Tris-HCl pH 6.8, 4% (w/v) SDS, 0.02% (w/v) bromophenol blue, 200 mM DTT, 20% glycerol). Based on the absorbance measured at 280 nm, equal amounts of samples were loaded on 15% SDS-PAGE and then transferred to PVDF membrane (Cytiva) for 1 h at 4°C using a tank-blot system (Bio-Rad Laboratories). Afterwards, the membrane was washed three times with TBS buffer (20 mM Tris-HCl pH 8.0, 250 mM NaCl) upon shaking at 20°C for 5 min. Blocking was conducted in TBS-T buffer (20 mM Tris-HCl pH 8.0, 250 mM NaCl, 0.1% Tween-20) supplemented with 10% skimmed milk for 1 h at 20°C. The membrane was rinsed and washed three times at 20°C with TBS-T followed by washing in TBS. Rabbit serum with polyclonal antibodies raised against the purified LipA (Speedy 28-Day program, Kaneka Eurogentec S.A.) were added in 1:2,000 dilution in TBS-T with 2% BSA and incubated overnight at 4°C. Afterwards, the membrane was rinsed and washed three times at 20°C with TBS-T followed by washing in TBS. Secondary antibodies conjugated with horseradish peroxide (goat/anti-rabbit; Sigma A545) were added in 1:20,000 dilution in TBS-T with 2% BSA and incubated for 1 h at 20°C. The membrane was rinsed and washed three times at 20°C with TBS-T followed by washing in TBS. The blot was developed with Westar C Ultra 2.0 chemiluminescent substrate (Cyanagen) and imaged on Amersham Imager 680RGB (Cytiva).

## Results

### LipA aggregation is mediated by the lid domain

To investigate folding and activation of the lipase *in vitro*, LipA lacking the N-terminal signal peptide was heterologously overexpressed in *E. coli*. The overexpression resulted in formation of inclusion bodies consisting nearly exclusively of LipA, and the protein was isolated in the urea-denatured state (Figure 1B). The denatured LipA is a relevant mimetic of the protein that enters the periplasm as an unfolded polypeptide chain, and it was used for the further analysis. To examine whether the recombinant protein may be refolded into its functional form, LipA was diluted into the urea-free low-salt TGCG buffer (5 mM Tris pH 9.0, 5 mM glycine, 1 mM CaCl_2_ and 5% glycerol) and incubated in the presence of the foldase chaperone LipH. The enzymatic activity of LipA was assessed then via measuring the hydrolysis of a model substrate, *para*-nitrophenyl-butyrate (*p*NPB): Accumulation of the product, *p*-nitrophenolate, was followed colorimetrically until reaching the signal saturation, and the activity of the lipase A was quantified (Figures 1C,D). In the absence of LipH, the signal remained at the level of the autohydrolysis of *p*NPB, so the hydrolytic activity of LipA was not induced. Thus, the recombinant LipA could be folded *in vitro*, and LipH was required for activation of LipA.

**FIGURE 2:**
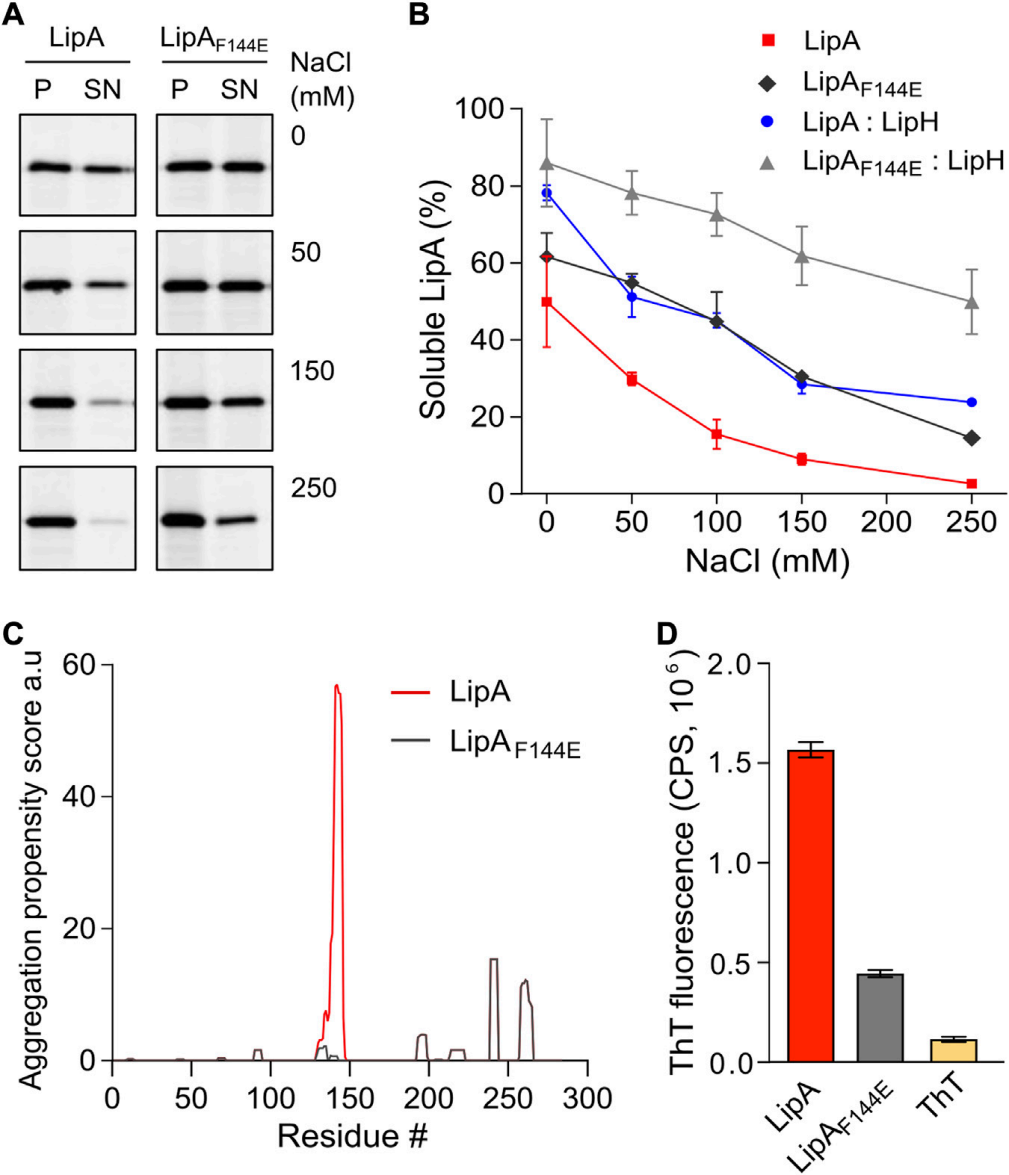
LipA aggregation propensity is sensitive to the environment and the structure of the lid domain. **(A)** SDS-PAGE of the aggregated and soluble LipA variants separated into the pellet (P) and the supernatant (SN) fractions, respectively, upon centrifugation. The concentrations of NaCl in the buffer are indicated. For the in-gel fluorescence detection, the lipases were labelled with fluorescein-5-maleimide. **(B)** Quantification of the soluble LipA at various conditions. The fraction of the soluble wild-type LipA and the mutant LipA_F144E_ at various salt concentrations and, optionally, in presence of LipH was calculated from SDS-PAGE (panel A). The assays were performed in technical triplicates, the mean values and SD are shown. **(C)** Sequence-based prediction of LipA aggregation propensity by TANGO algorithm (Fernandez-Escamilla et al., 2004). Profiles for the β-aggregation propensity scores of the wild-type LipA (red) and the mutant LipA_F144E_ (grey) indicate the aggregation-prone regions within the polypeptide chain. The aggregation propensity of the wild-type LipA is dominated by the α-helix 5 that forms the lid domain. **(D)** LipA aggregation is associated with the formation of β-structured amyloid-like aggregates. The intensity of the amyloid-sensitive ThT fluorescence at 485 nm was measured in the presence of either the wild-type LipA (red), the mutant LipA_F144E_ (grey), or the dye alone (yellow). The assays were performed in technical triplicates, the mean values and SD are shown.

Notably, if LipA was incubated in the urea-free buffer prior to adding the foldase LipH, its hydrolytic activity was substantially reduced, as the amount of *p*-nitrophenolate generated in the first 15 min of the reaction decreased three-fold (Figures 1C,D). We speculated that the lipase underwent aggregation/misfolding in the absence of the foldase LipH, causing the loss of activity. Indeed, we observed a rapid increase in light scattering once the lipase was incubated in absence of urea (Supplementary Figure S1A), and approx. 50% of LipA could be sedimented upon mild centrifugation at 21000 g (Figure 2A). The sedimentation was enhanced upon increasing the ionic strength of the solution, reaching 70% and 95% in the presence of 50 and 150 mM NaCl, respectively (Figure 2B) and abundant large particles were observed by negative-stain electron microscopy (Supplementary Figure S1B). The stoichiometric amount of LipH stabilised the lipase, although the efficiency decreased at elevated salt concentrations and the hydrolytic activity of the lipase reduced (Figure 2B and Supplementary Figure S1C). Since LipA:LipH binding is primarily driven by electrostatic interactions (El Khattabi et al., 2000; Pauwels et al., 2006, 2012), the affinity of the complex was likely reduced at the high ionic strength and LipA aggregation became a dominant pathway even in the presence of the chaperone.

Based on its amino acid sequence, the α-helix 5 known as the “lid domain” of LipA is predicted to be the primary aggregation-prone region within the protein, with a high propensity to misfold into β-strands (Figure 2C, red line) (Fernandez-Escamilla et al., 2004). Previous studies on a homologous lipase demonstrated that a point mutation Phe to Glu within the lid domain diminished the aggregation propensity and rendered an aggregation-resistant protein (Rashno et al., 2018). Based on the structure homology, we recognised an identically positioned phenylalanine residue within *P. aeruginosa* PAO1 LipA at position 144, where the point mutation to glutamate could suppress the lipase misfolding (Figure 2C, black line). To examine the effect of the mutation on the lipase folding and activity, the mutant LipA_F144E_ was overexpressed and isolated in its urea-denatured state. In the presence of LipH, LipA_F144E_ displayed the enzymatic activity, indicating that the mutation did not hinder the chaperone-mediated folding (Figures 1C,D). Indeed, the lid domain is not involved in LipA:LipH contacts (Pauwels et al., 2006), and the mutated residue is oriented toward the solvent and does not belong to the moiety of the catalytic site (Figure 1A).

To examine whether LipA_F144E_ is indeed more resistant against aggregation than the wild-type lipase, its solubility was probed over a range of salt concentrations. The point mutation had a notable effect on the protein stability: Nearly 50% of LipA_F144E_ remained soluble in the presence of 100 mM NaCl, where the soluble fraction of the wild-type LipA did not exceed 15% (Figure 2B), and the activity of the refolded lipase mutant was weakly affected by the ionic strength (Supplementary Figure S1C). As the aggregated lipase tends to form β-sheet-rich structures and amyloids (Rashno et al., 2018), we further examined this propensity for the wild-type LipA and LipA_F144E_ mutant. To do so, we measured the fluorescence intensity of the dye thioflavin T (ThT), which fluorescence increases manifold upon binding to β-strands characteristic to amyloids (Biancalana & Koide, 2010). Once the dye was added to the wild-type lipase in the TGCG buffer supplemented with 20 mM NaCl, its fluorescence intensity increased 10-fold, suggesting that β-stranded structures were formed even at the low salt concentration (Figure 2D and Supplementary Figure 1D). In contrast, the ThT fluorescence was only modestly affected in the presence of LipA_F144E_, increasing 3-fold above the signal of free ThT, in agreement with the reduced aggregation of the mutant.

As both the mutation within the lid domain of LipA and the LipA:LipH assembly favoured the soluble state of the lipase, we questioned whether both cases involve the same stabilisation mechanism. To address this, the solubility of the wild-type LipA and LipA_F144E_ in the presence of the foldase was compared (Figure 2B). Strikingly, the solubility of the mutant LipA_F144E_ was further enhanced by LipH: At elevated salt concentrations, the fraction of the soluble LipA_F144E_ exceeded approx. two-fold that of the wild-type LipA, so even at 250 mM NaCl the major fraction of LipA_F144E_:LipH remained soluble. Conclusively, the effects of the mutation within the lid domain and the lipase:foldase binding were additive, and distinct mechanisms must have contributed to the lipase stabilisation.

### Characterisation of periplasmic chaperones of *P. aeruginosa*

The extensive aggregation of the wild-type LipA observed already at moderate salt concentrations is a potential challenge for its timely interactions with the stabilising foldase LipH, especially under high-salinity conditions within cystic fibrosis patients’ sputum (Grandjean Lapierre et al., 2017). As highly abundant periplasmic chaperones may facilitate folding and secretion of several recombinant proteins, including a recombinantly expressed lipase of *Burkholderia* (Purdy et al., 2007; Jarchow et al., 2008; Narayanan et al., 2011; Entzminger et al., 2012; Dwyer et al., 2014), we speculated that the chaperones of *P. aeruginosa* may recognize LipA and protect it from aggregation prior interactions with the specific foldase LipH.

**FIGURE 3:**
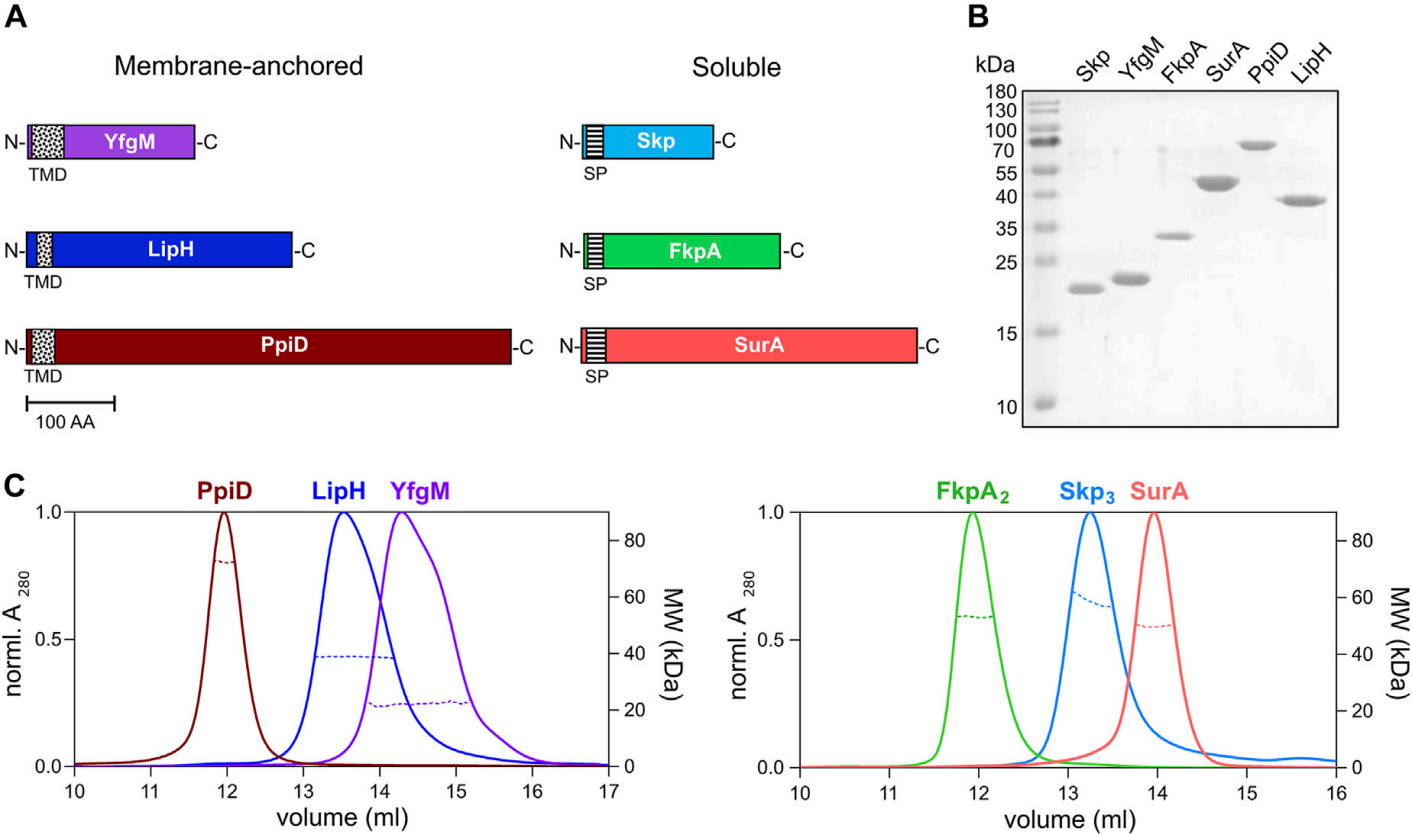
Periplasmic chaperones of *P. aeruginosa* PAO1. **(A)** Schematic overview of the periplasmic chaperones of *P. aeruginosa* PAO1. The predicted transmembrane domains (TMD, black-dotted) and the signal peptides (SP, black-ruled) were removed to express the soluble chaperones. Scale bar: 100 amino acid residues (AA). **(B)** SDS-PAGE of the isolated periplasmic chaperones of *P. aeruginosa*. **(C)** SEC-MALS analysis of the periplasmic chaperones to determine the oligomeric states in solution. Solid traces represent the absorbance at 280 nm, and dashed traces indicate the molecular weights (*y*-axis on the right) calculated from the light scattering. The analysis reveals dimers of FkpA (FkpA_2_) and trimers of Skp (Skp_3_), while SurA, YfgM, PpiD and LipH are monomeric in solution.

To characterize LipA:chaperone interactions in a well-defined *in vitro* system, the primary soluble chaperones Skp, FkpA and SurA lacking their signal peptides (Figure 3A), as well as the periplasmic domains of YfgM and PpiD of *P. aeruginosa* PAO1 were heterologously expressed and purified from *E. coli.* The apparent molecular weights of the chaperones observed in SDS-PAGE agreed with the values calculated for individual protomers (18 kDa for Skp, 20 kDa for YfgM, 27 kDa for FkpA, 47 kDa for SurA and 67 kDa for PpiD; Figure 3B). To scrutinise the oligomeric states of the isolated chaperones, their native masses were determined by size exclusion chromatography combined with multi-angle light scattering analysis (SEC-MALS, Figure 3C). The experiment revealed monomers of SurA (determined molecular weight of 49.6 ± 0.6 kDa), dimers of FkpA (53.2 ± 0.9 kDa; FkpA_2_), and trimers of Skp (58.6 ± 0.7 kDa; Skp_3_), in agreement with the known structures of homologs from *E. coli* (Bitto & McKay, 2002; Saul et al., 2004; Walton & Sousa, 2004). The periplasmic domains of both YfgM and PpiD were found to be monomers, with molecular masses of 22.2 ± 0.4 kDa and 72.2 ± 0.2 kDa, respectively. The periplasmic chaperone domain of LipH appears to be monomeric in solution (determined molecular weight 39.4 ± 0.3 kDa). The attempts to analyse the molecular mass of either wild-type LipA or LipA_F144E_ were not successful, as the hydrophobic protein was possibly interacting with the column matrix and could not be eluted with aqueous buffers.

### The chaperone Skp prevents LipA from aggregation

**FIGURE 4:**
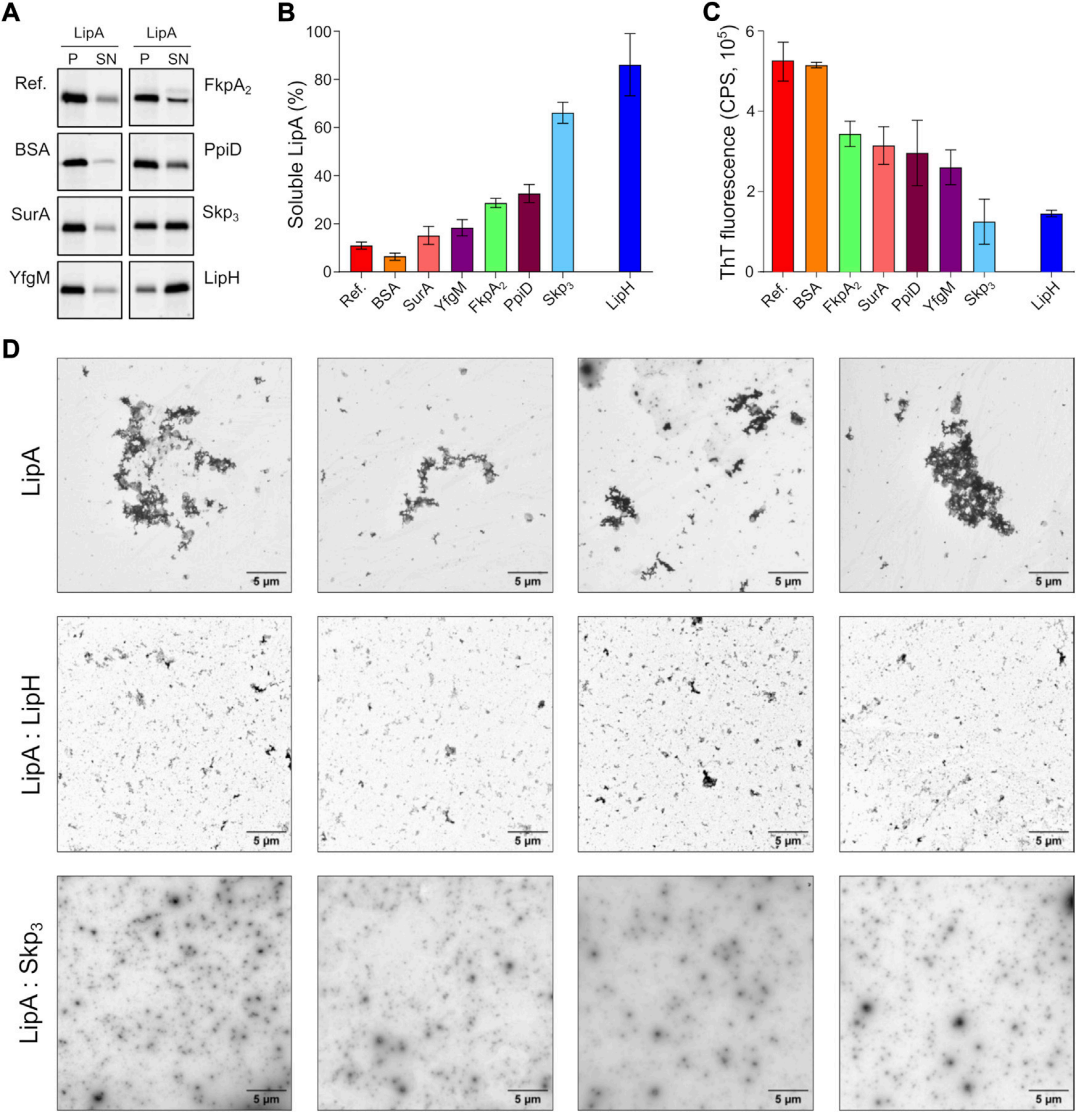
The periplasmic chaperone Skp rescues LipA from aggregation. **(A)** LipA solubility in the presence of the periplasmic chaperones. The aggregated and soluble LipA fractions are separated in pellet (P) and supernatant (SN) fractions in the sedimentation assay and visualized on SDS-PAGE via in-gel fluorescence. The supplemented chaperones are indicated. Bovine serum albumin (BSA) was used as a negative control. LipA in absence of chaperones is indicated as “Ref.”. **(B)** Quantification of the soluble LipA in the presence of the periplasmic chaperones from SDS-PAGE (panel A). The assays were performed in technical triplicates, the mean values and SD are shown. **(C)** The periplasmic chaperones suppress formation of LipA β-structured aggregates. ThT fluorescence was recorded for the wild-type LipA in the presence of the periplasmic chaperones, and the fluorescence measured for the chaperones alone was subtracted. The assays were performed in technical triplicates, the mean values and SD are shown. **(D)** LipA aggregation in presence and absence of LipH (LipA:LipH) or Skp (LipA:Skp_3_) visualized *via* negative-stain TEM. Scale bars are indicated. The relatively low contrast repeatedly observed in LipA:Skp_3_ samples was attributed to the high concentration of the chaperone (45 µM of monomeric Skp).

The propensity of the individual chaperones to prevent LipA aggregation was first evaluated by the sedimentation analysis. To mimic the natural abundance of SurA, FkpA_2_ and Skp_3_ in the bacterial periplasm, as described for the model organism *E. coli* (Schmidt et al., 2016; Tsirigotaki et al., 2017), and to ensure the uniform lipase:chaperone molar ratio of 1:10, 0.5 µM LipA and 5 µM of chaperones were used in the experiments, corresponding to 10 µM of FkpA and 15 µM Skp monomers. The salt concentration was set to 150 mM NaCl, which closely matches the salinity of the sputum environment (Grandjean Lapierre et al., 2017). In the absence of the chaperones, only 10% of LipA was found in the soluble form, and the value increased moderately in the presence of either SurA or FkpA_2_ (solubility of 15% and 29%, respectively; Figures 4A,B). In contrast, Skp_3_ demonstrated prominent stabilisation of LipA, as the soluble fraction reached 66%. In the presence of the lipase-specific foldase LipH, more than 80% LipA was found soluble (LipA:LipH molar ratio 1:1).

The membrane-anchored proteins PpiD and YfgM are the recently described chaperones associated with the SecYEG translocon of *E. coli* (Matern et al., 2010; Götzke et al., 2014). Being proximal to the translocon, the peptidyl prolyl isomerase PpiD and the tetratricopeptide repeat-containing protein YfgM of *P. aeruginosa* may interact with LipA at the early stage of its translocation into the periplasm and mediate the handover to the membrane-anchored LipH. Similar to the soluble chaperones above, the potential effects of the periplasmic domains of PpiD and YfgM on LipA aggregation were investigated. YfgM did not stabilize LipA, as the soluble fraction remained below 20% (Figures 4A,B). This lack of the holdase activity is in agreement with earlier experiments, where luciferase and the precursor of OmpC were tested as putative YfgM clients (Götzke et al., 2014). In the presence of PpiD, the soluble fraction of LipA increased up to 34%, which matched closely the value measured for another peptidyl prolyl isomerase, FkpA_2_. Overall, the sedimentation assay identified the non-specific soluble chaperone Skp as a potent stabilising factor of the lipase.

Next, we questioned whether the chaperones might be capable of preventing the assembly of β-sheet-rich aggregates of LipA. To probe this putative effect, ThT fluorescence was measured for LipA in the presence of individual chaperones and corrected to the values of the chaperones alone (Supplementary Figure S2). Although most of the chaperones had moderate to low effect on LipA solubility in the sedimentation assay (Figure 4B), they all could reduce the ThT fluorescence at least by 40%, as compared to LipA alone or LipA in the presence of bovine serum albumin (BSA) (Figure 4C). These values reflect the partial ability of chaperones to prevent formation of β-sheet-rich aggregates by LipA. Notably, the most prominent effect was observed again for Skp_3_, as the β-sheet-specific ThT signal in the presence of the chaperone was reduced by approx. 75%. Similar low ThT fluorescence intensity was recorded in the presence of LipH.

Finally, negative-stain electron microscopy was used to visualize LipA aggregation. At the elevated ionic strength and in the absence of urea, LipA readily formed micrometer-sized aggregates, commonly assembled in larger clusters (Supplementary Figure S1B and Figure 4D). The sample morphology was altered when either LipH or Skp_3_ were added at the molar ratios of 1:3 and 1:5, respectively, so only sub-micrometer featureless particles could be observed, suggesting reduced aggregation propensity. Thus, we concluded that both Skp and LipH offer protection from the assembly of aggregates, despite different mechanisms of interaction with the lipase.

### The periplasmic chaperones modulate the activation of LipA

**FIGURE 5:**
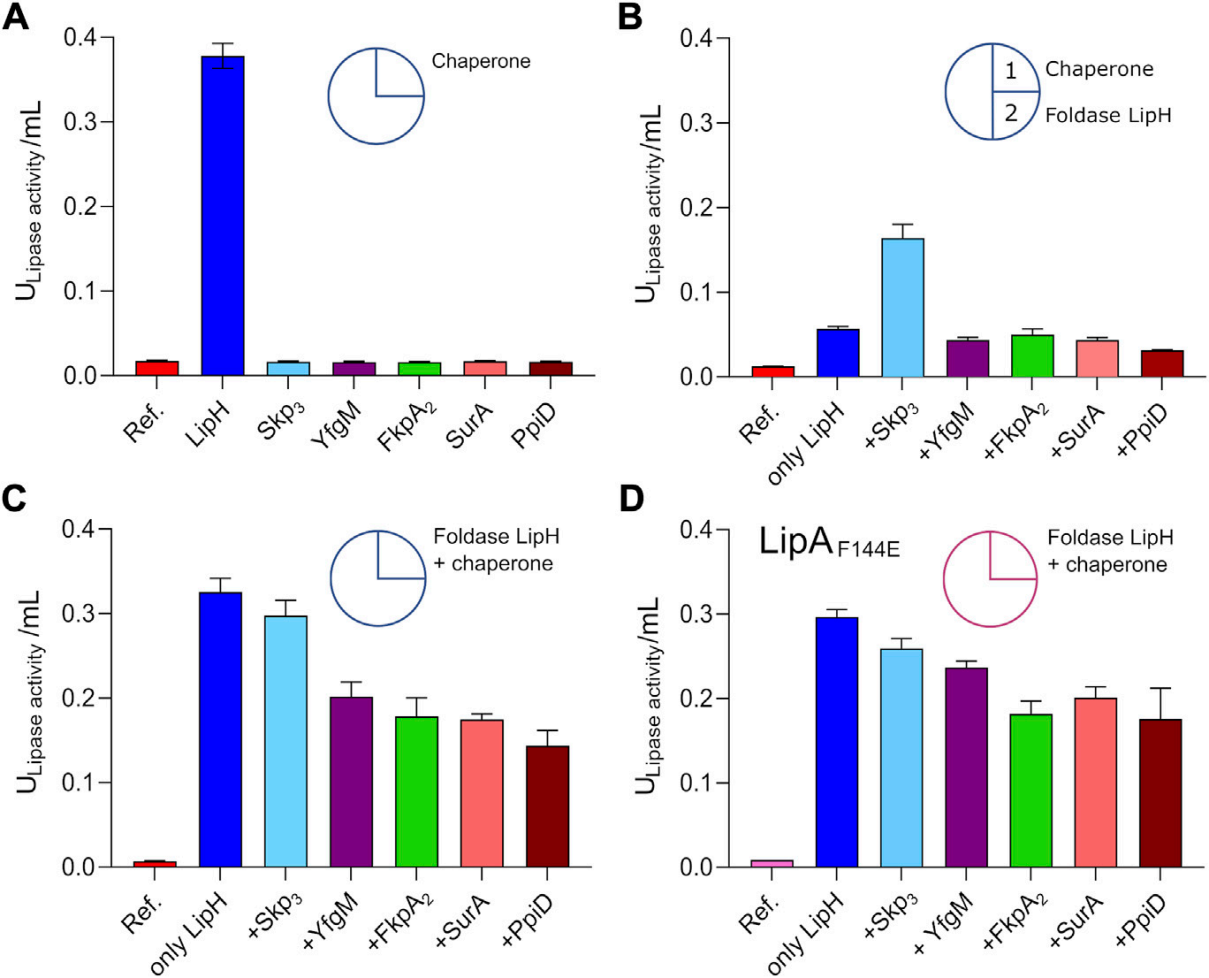
Interplay of periplasmic chaperones upon the lipase activation. The lipase activity against *p*NPB over the first 15 min of the hydrolysis reaction is shown in bars. Circle scheme describes the incubation steps. LipA activity in absence of chaperones is indicated as “Ref.”. **(A)** Incubation of the wild-type LipA with either the foldase LipH or general periplasmic chaperones in the TGCG buffer. **(B)** Initial incubation of the wild-type LipA with general periplasmic chaperones at 100 mM NaCl (1) followed by incubation with LipH (2). **(C)** Simultaneous incubation of the wild-type LipA with LipH and periplasmic chaperones in 50 mM NaCl, 1 mM CaCl_2_, 10% glycerol (v/v) and 20 mM Tris-HCl pH 8.0. **(D)** Simultaneous incubation of the mutant LipA_F144E_ with LipH and periplasmic chaperones in 50 mM NaCl, 1 mM CaCl_2_, 10% glycerol (v/v) and 20 mM Tris-HCl pH 8.0. The assays were performed in technical triplicates, the mean values and SD are shown.

The interactions of the periplasmic chaperones with LipA and their anti-aggregation effects may affect the functionality of the lipase, e.g., by directly altering its folding pathways and/or by competing with the specific foldase LipH. None of the general periplasmic chaperones alone rendered the active lipase, as no enzymatic hydrolysis of *p*NPB was observed in the absence of LipH (Figure 5A). To examine the potential competition between the chaperones, we focused on the LipH-mediated activation of LipA in the presence of the general periplasmic chaperones, as it occurs in living cells. The wild-type LipA was pre-incubated either alone or in presence of 5-fold excess of Skp_3_, YfgM, FkpA_2_, SurA or PpiD under conditions of elevated ionic strength (100 mM NaCl, 1 mM CaCl_2_ and 20 mM Tris-HCl pH 8.0) sufficient to induce the lipase aggregation. After the pre-incubation phase, the foldase LipH was added to activate LipA, and the resulting hydrolytic activity against *p*NPB was determined (Figure 5B). The conditions of the pre-incubation phase had an evident influence on LipA functionality: Once LipA was incubated without chaperones, its activity was suppressed to a large extent (blue bars in Figure 5A vs Figure 5B), in agreement with the documented aggregation of the lipase. The pre-incubation in the presence of SurA, FkpA_2_, YfgM or PpiD chaperones either did not enhance or even further decreased LipA activity (Figure 5B). Differently though, pre-incubation of LipA with Skp_3_ resulted in prominent stimulation of the substrate hydrolysis, in agreement with the anti-aggregation effect of the chaperone.

The reduced LipA activity in the presence of FkpA_2_, SurA, YfgM or PpiD correlated with their poor propensity to prevent LipA aggregation, but also suggested the interference with the LipH-mediated activation, potentially by competing for the lipase binding. To examine this scenario, both, the general chaperones and LipH, were added to LipA simultaneously at the LipA:LipH:chaperone molar ratio of 1:1:5, so the lipase activity was dependent on the folding/aggregation balance and the competition between the chaperones for the lipase. In the presence of either SurA, FkpA_2_, YfgM or PpiD, the activity of LipA dropped by 30–50% (Figure 5C), while only minor, below 10% inhibition of substrate hydrolysis was observed for LipA:LipH:Skp_3_ sample. Finally, to scrutinise the effect of the chaperone competition, the assay was conducted using the aggregation-resistant LipA_F144E_ mutant (Figure 5D). When either SurA, FkpA_2_, YfgM or PpiD were present in addition to LipH, the lipase activity reduced by 20–30%, so we concluded that the chaperones interfered with the LipH:LipA assembly. The effect of Skp_3_ on LipH-mediated activation was minimal, so the chaperone allowed efficient LipH:LipA interactions, while being capable of preventing LipA aggregation.

**FIGURE 6:**
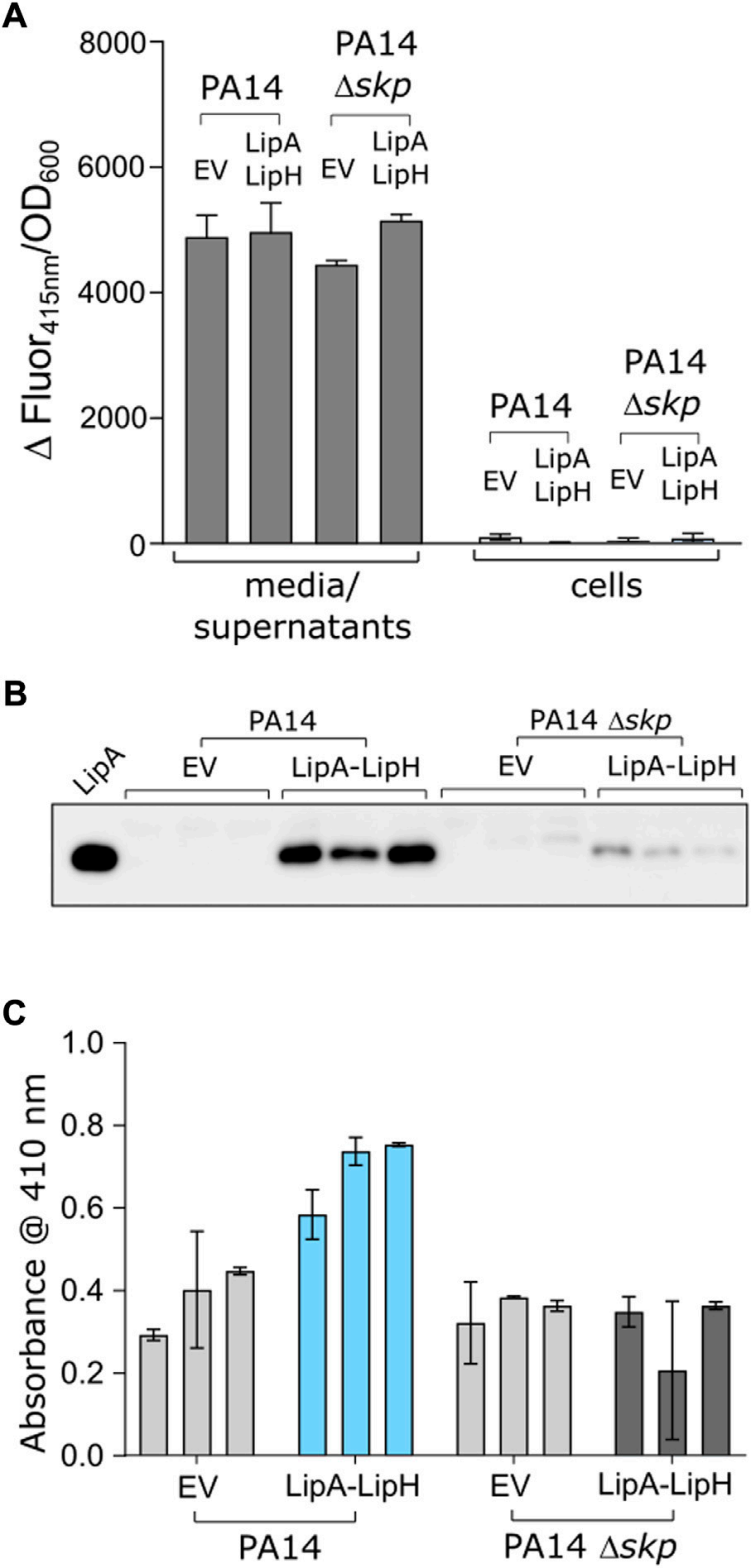
Deletion of *skp* gene in *P. aeruginosa* abolishes LipA secretion. **(A)** The functionality of *P. aeruginosa* T2SS is not affected by deletion of *skp* gene. Hydrolysis of Abz-Ala-Gly-Leu-Ala-*p*-nitro-benzyl-amide substrate by T2SS-dependent elastase was monitored as an increase in fluorescence at 415 nm in the cell-free media and in cells of *P. aeruginosa* strains PA14 and PA14 Δ*skp* carrying the empty vector (EV, pGUF) and LipA-LipH expression vector, as described. **(B)** Western blot against the secreted LipA in *P. aeruginosa*. Wild-type PA14 and *skp* null-mutant (PA14 Δ*skp*) strains were transformed with either an empty vector (“EV”) or a plasmid encoding for lipA-lipH operon (“LipA-LipH”). Recombinantly expressed and purified LipA was used as a reference (left lane). The complete western blot is provided in Suppl. Figure 3A. **(C)** Lipolytic activity against *p*NPB measured in the extracellular media of *P. aeruginosa* PA14 strains described in the panel E.

We questioned then whether the observed favourable effect of Skp_3_ on LipA solubility and activation also contributes to the lipase biogenesis *in vivo*. To examine this possibility, we analysed LipA secretion in the highly virulent *P. aeruginosa* strain PA14, where *skp/hlpA* gene was optionally knocked out (Klein et al., 2019). In contrast to the deletion of the essential *surA* gene, knock-out of *skp* does not have impact on the outer membrane integrity and so may have minor pleiotropic effect in cells (Klein et al., 2019). To verify that the *skp* deletion did not affect the T2SS assembly, we examined the activity of the elastase LasB, another secretory enzyme that utilizes the T2SS route. No significant change in hydrolysis of a model substrate was observed between various samples (Figure 6A), so we concluded that the absence of Skp chaperone was not detrimental for T2SS integrity and functionality. However, detection of the secreted lipase in the cell-free culture supernatant by immunoblotting revealed a drastic decrease in the amount of LipA upon deletion of Skp (Figure 6B and Supplementary Figure S3A). In agreement with the abolished secretion of LipA, the primary lipase of *P. aeruginosa* (Figure 6C), the total esterase activity measured for the cell-free media decreased approx. 2-fold, and the remaining activity could be related to other enzymes, such as the lipase LipC (Rosenau et al., 2010). Since only the mature form of LipA was detected in the western blot, and no oxidase activity was measured in the media for any of the samples (Supplementary Figure S3B), significant cell lysis could be excluded from consideration, so the differences in LipA abundance and the lipolytic activity must have originated from the altered secretion levels, likely dependent on LipA:Skp_3_ interactions.

### Trimeric Skp of *P. aeruginosa* acquires multiple conformations in solution

With the interest in the potent effect of Skp on LipA folding and secretion, we set out to shed more light on the functional mechanism of the chaperone. The well-studied Skp homolog from *E. coli* (*Ec*Skp_3_) forms a “jellyfish”-like trimer that resembles the arrangement of prefoldin-type chaperones of mitochondria and archaea (Korndörfer et al., 2004; Walton & Sousa, 2004). Each protomer consists of the distinct “head” and “arm” domains. The “head” domain of *Ec*Skp_3_ is predominantly formed by β-sheets which drive the oligomerization, and the “arm” domain is a helical section forming a hairpin extension. The client-binding pocket between the “arm” domains is constituted by all three protomers. The crystal structures and small-angle X-ray scattering (SAXS) experiments predicted a broad range of conformations for the *Ec*Skp trimer in its apo-state in solution, where three major states termed “closed”, “intermediate” and “open” were described (Holdbrook et al., 2017).

**FIGURE 7:**
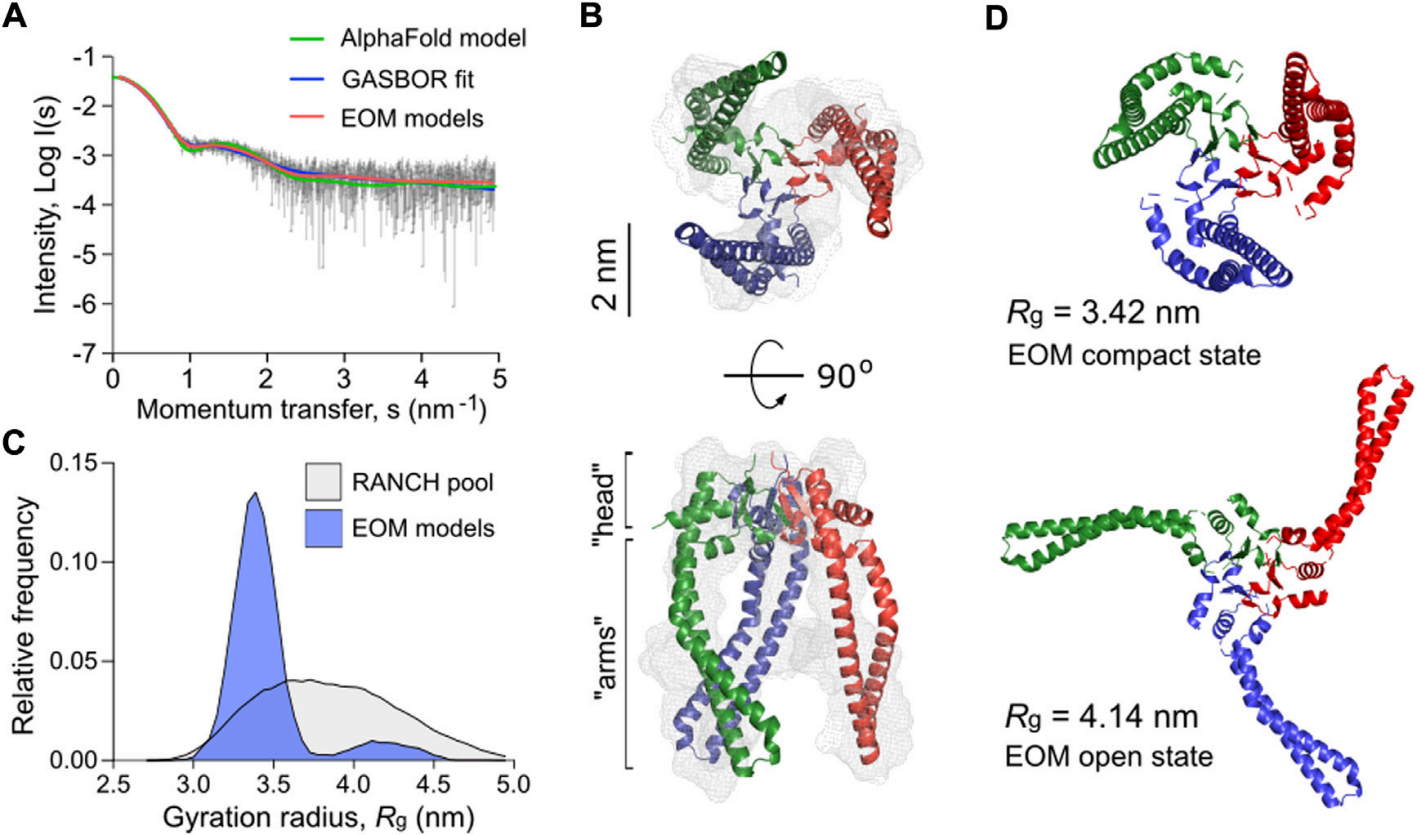
*P. aeruginosa* Skp forms a flexible trimer in solution. **(A)** Experimental SAXS data curve is shown in grey dots with error bars. The intensity (log *I*) is displayed as a function of the momentum transfer (s). The intensity patterns of Skp_3_ models derived from GASBOR (blue), AlphaFold (green), and EOM (red) algorithms are overlaid. **(B)** The experimental SAXS envelope of *P. aeruginosa* Skp (grey mesh) calculated using GASBOR algorithm reflects the trimeric structure of the chaperone. The structural model of Skp_3_ is superimposed with the SAXS envelope, with each protomer coloured individually (red, green, blue). The “head” and “arm” structural domains of Skp are indicated. The scale bar is shown on the side. **(C)** The distribution of Skp_3_ conformations differing by their gyration radii (*R*_g_) calculated by EOM (blue). The original RANCH pool based on 10000 random models is shown in grey. **(D)** Representative conformations of *P. aeruginosa* Skp_3_ calculated by EOM in the compact (*R*_g_ of 3.42 nm, *D*_*max*_ of 9.73 nm; fraction ∼0.62) and the open (*R*_g_ of 4.14 nm, *D*_*max*_ of 13.63 nm; fraction ∼0.12) states.

Mature Skp of *P. aeruginosa* shares 22% sequence identity with *Ec*Skp and contains three unique proline residues per protomer, including two prolines in the predicted “head” domain and one in each helical “arm”, which may alter the flexibility of the chaperone (Supplementary Figure S4). Thus, we employed SAXS to experimentally assess the conformational ensemble of *P. aeruginosa* Skp in solution (Figure 7A, Supplementary Figures S5A–D and Supplementary Table S1). In agreement with SEC-MALS data (Figure 3), Skp was found to be a trimer. The characteristic “jellyfish”-like shape with the “head” and “arm” domains of the trimer could be recognized in the *ab initio* reconstitution of the oligomer structure using the GASBOR program (Svergun et al., 2001) (χ^2^ = 1.016) (Figures 7A,B). A structural model of *P. aeruginosa* Skp_3_ built by AlphaFold 2 algorithm (Evans et al., 2021; Jumper et al., 2021) could be fitted into the experimental SAXS envelope (Figure 6B). However, the measured radius of gyration (*R*_g_) of 3.50 nm for the averaged SAXS data is slightly higher than *R*_*g*_ of 3.32 nm calculated via CRYSOL for the modelled structure (χ^2^ = 1.28) (Svergun et al., 1995), so Skp_3_ of *P. aeruginosa* adopts a more open conformation.

The SAXS data also revealed some flexibility in Skp_3_ of *P. aeruginosa*, as previously observed for the *Ec*Skp_3_ homolog (Holdbrook et al., 2017). To characterize this conformational space, Ensemble Optimization Method (EOM) was applied (Bernadó et al., 2007; Tria et al., 2015). EOM generates a pool of sequence- and structure-based models (RANCH models; Figure 7C and Supplementary Figures S5E, S5F), and the implemented genetic algorithm GAJOE (Genetic Algorithm Judging Optimisation of Ensembles) selects an ensemble until an optimal fit to the experimental SAXS curve is reached. The selected models describe best the experimental SAXS data, and likely represent the most frequent conformations of the studied protein (Figure 7B). To model the Skp_3_ dynamics by EOM, the “head” domain within the trimer was fixed and the helical “arms” were allowed moving to fit the SAXS data. As a result, the selected ensemble matched the experimental SAXS curves with χ^2^ = 0.954 (Figure 6A, EOM models). The distribution of the models showed two primary states, resembling those reported for *Ec*Skp_3_ (Holdbrook et al., 2017): The “compact” state with *R*_g_ of 3.42 nm and maximal dimensions (*D*_max_) of 9.73 nm, which accounts up to 62% of the population, and the “open” state (*R*_g_ = 4.14 nm, *D*_max_ = 13.63 nm; 12% occupancy) (Figure 7D). While the dimensions of the open state were nearly identical for both Skp_3_ homologs, the compact state of Skp_3_ of *P. aeruginosa* was wider than the “intermediate” conformation of *Ec*Skp_3_ (*R*_g_ of 3.42 vs. 3.2 nm), and no “closed” state was observed for Skp_3_ of *P. aeruginosa.* Nevertheless, the ability of Skp_3_ of both *E. coli* and *P. aeruginosa* to undergo a large conformational change from a compact to a widely open state suggests that the proteins follow the same functional dynamics*.*

### Two copies of Skp_3_ are required to stabilize soluble LipA *via* apolar contacts

The documented clients of Skp in the bacterial periplasm span a broad range of molecular masses, from 19 to 89 kDa, and they belong to various classes, both membrane-associated and globular proteins (Qu et al., 2007; Schiffrin et al., 2016). Though the stimulatory effect of Skp_3_ on secretion of a heterologous bacterial lipase has been previously demonstrated, it remained obscure whether the stimulation was indeed a result of direct client:chaperone interactions (Narayanan et al., 2011). Our biochemical and *in vivo* data substantiates the view that Skp_3_ is a potent chaperone that prevents the lipase aggregation in *P. aeruginosa*, and here we set out to characterize LipA:Skp_3_ interactions in detail.

**FIGURE 8:**
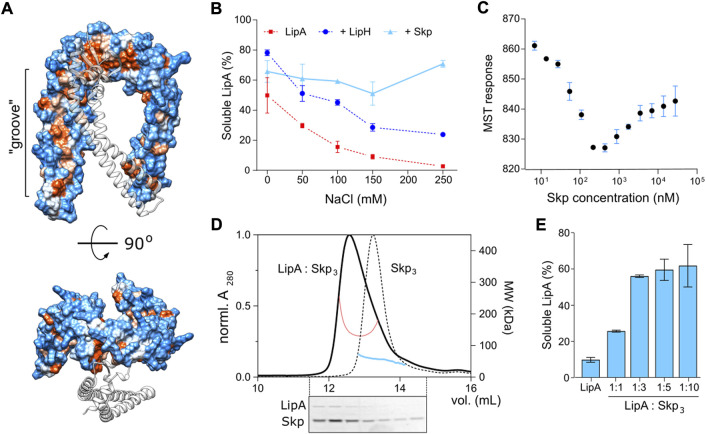
Characterisation of LipA:Skp_3_ interactions. **(A)** Surface hydrophobicity plot of *P. aeruginosa* Skp_3_. The hydrophobic groove along the “arm” domain is indicated. Polar/charged residues are shown in blue, apolar in orange. One subunit within the Skp trimer is shown as a transparent ribbon for clarity. **(B)** Skp supports the solubility of LipA over a broad range of ionic strength. The soluble fraction of the wild-type LipA in presence of Skp (light blue) was determined from SDS-PAGE after the sedimentation assay at various salt concentrations. The assays were performed in technical triplicates, the mean values and SD are shown. Salt-dependent aggregation of LipA alone and LipA in presence of LipH are indicated in red and dark blue, respectively, and correspond to those in Figure 2B. **(C)** LipA:Skp_3_ interactions manifest biphasic behaviour. Microscale thermophoresis of LipA_F144E_:Skp interactions was performed using fluorescently labelled LipA_F144E_ and Skp titrations (monomers concentration indicated). The high-affinity interaction at 20 nM Skp may be assigned to LipA binding a single chaperone trimer, followed by recruitment of the second chaperone with a lower affinity. The assay was performed in technical triplicates, the mean values and SD are shown. **(D)** SEC-MALS of the LipA_F144E_:Skp_3_ complex and Skp_3_ alone. Normalized UV absorbance of LipA_F144E_:Skp_3_ (solid black line) and Skp_3_ (dotted black line), and the corresponding molecular weights are indicated. Below: SDS-PAGE of the LipA_F144E_:Skp_3_ eluate. **(E)** Solubility of the lipase is dependent of the LipA:Skp_3_ ratio. The soluble fraction of the wild-type LipA was determined from SDS-PAGE after the sedimentation assay at various LipA:Skp_3_ ratios. The assays were performed in technical triplicates, the mean values and SD are shown.

Firstly, we questioned whether the interaction of *P. aeruginosa* Skp_3_ with LipA is indeed of hydrophobic nature, as each of the “arms” within the Skp trimer has an apolar side (Figure 8A) that may bind unfolded and/or misfolded clients. As shown for the LipA:LipH complex, elevated ionic strength of the aqueous solution severely destabilizes the hydrophilic binding interface, so the released LipA irreversibly aggregates (Figure 2B). Hydrophobic interactions, in contrast, should be insensitive to the ionic strength and may facilitate the chaperoning at high salt concentrations. The solubility of the wild-type LipA in the presence of Skp_3_ at the molar ratio 1:10 was examined over a range of NaCl concentrations via the sedimentation assay (Figure 8B). Different to LipA alone and LipA in the presence of LipH, no decay in the soluble fraction was observed when Skp_3_ was present. At the highest examined concentration of 250 mM NaCl, Skp_3_ ensured the lipase solubility of approx. 70%, while the chaperone-free LipA was nearly completely aggregated, and only 30% of LipA was found soluble in the presence of LipH. This observation implied that the lipase is recognized via non-polar contacts, likely associated with the unfolded state.

Next, we set out to determine the binding affinity of Skp to LipA. Earlier studies of Skp interactions with its most abundant clients, OMPs, provided the affinity estimates at low-nanomolar to low-micromolar scales for OMP:Skp_3_ complexes formed at 1:1 and 1:2 stoichiometry, respectively (Qu et al., 2007; Schiffrin et al., 2016; Mas et al., 2020; Pan et al., 2020), while the interactions with soluble proteins have not been addressed. For the analysis here, we employed microscale thermophoresis (MST) and used the aggregation-resistant mutant LipA_F144E_ as the fluorescently labelled component. LipA_F144E_-FM was mixed with Skp ranging from 4.5 nM to 37 µM (concentration of Skp monomers), and the MST response of LipA_F144E_-FM, i.e., its mobility within the micrometer-sized temperature gradient, was analysed. Strikingly, LipA_F144E_ manifested a bimodal MST response upon increasing the Skp concentration that appeared as a V-shaped plot with a transition at approx. 300 nM Skp_1_ (100 nM Skp_3_) (Figure 8C). The data suggested two distinct modes of binding to the chaperone, where individual K_D_’s were ∼20 nM and 2 μM, and the values were in a good agreement with values measured for OMP:Skp_3_ interactions where either one or two Skp timers were involved (Qu et al., 2007; Schiffrin et al., 2016; Pan et al., 2020). Thus, we proposed that once Skp is present at micro-molar concentration, LipA may bind two chaperones simultaneously.

The LipA:Skp affinity measured in the MST experiments suggested that two copies of Skp trimer were recruited to stabilize the soluble lipase in the assays, described above (Figures 4, 5; monomeric Skp of 15 µM). To test the stoichiometry of the complex experimentally, SEC-MALS analysis was performed on LipA_F144E_:Skp mixture (molar ratio 1:3; Figure 8D). The elution peak demonstrated a prominent shift in comparison to Skp_3_ alone, and the estimated molecular mass of 139 kDa approached closely the calculated mass of the complex LipA:2*Skp_3_ (148 kDa). SDS-PAGE confirmed presence of both the chaperone and the lipase in the peak fractions, and the densitometry analysis of the corresponding bands suggested the excess of Skp (estimated LipA:Skp_3_ ratio 1:3). The remaining free LipA fraction was likely bound to the column resin, as we observed previously. Thus, we concluded that the relatively small protein LipA provided sufficient surface area for binding two copies of the trimeric Skp. To test, whether this stoichiometry was beneficial for the solubility of the lipase, we used the aggregation-prone wild-type LipA in the sedimentation assay where the LipA:Skp_3_ ratio was varied (Figure 8E). At the molar ratios of 1:10 and 1:3, above 70% of LipA could be rescued from aggregation by Skp_3_. Strikingly, the rescued fraction of LipA dropped dramatically when the ratio was reduced to 1:1 (monomeric Skp 1.5 µM), suggesting that a single Skp trimer was not capable of rescuing the client from aggregation/misfolding. An independent titration experiment was also performed to compare the anti-aggregase activity of Skp_3_ with other periplasmic chaperones (Supplementary Figure S6). In all three examined ratios Skp_3_ appeared as the most potent chaperone, although its propensity to rescue LipA was notably dependent on the concentration. Although the concentration of Skp_3_ in *P. aeruginosa* is unknown to date, it seems plausible that it is sufficiently high to ensure binding of LipA within the consolidated cavity of two Skp trimers. Further folding and activation of the lipase should depend on the hand-over and high-affinity specific interactions with the foldase LipH.

## Discussion

The protein fold is determined by its primary amino acid sequence, but the pathways followed by the polypeptide chain towards the functional structure commonly involve the assistance of chemical and proteinaceous chaperones. The chaperones steer their client dynamics along the energy landscape, facilitate the local and global folding/unfolding events, prevent off-pathway intermediates, and serve to disaggregate occasional misfolded structures (Hartl et al., 2011; Stull et al., 2018). The role of chaperones is of particular importance under stress factors, such as elevated temperature, or challenging environments, such as bacterial periplasm, where the protein folding should proceed under broadly varying conditions. Here, we demonstrate that the non-specific periplasmic chaperone Skp of the pathogenic bacterium *P. aeruginosa* efficiently protects the secretory lipase LipA from aggregation and suppresses its assembly into β-sheet-rich structures. The structural analysis of trimeric Skp in solution by SAXS reveals an ensemble of co-existing states. The interaction analysis of the LipA:Skp_3_ complex suggests that either one and two copies of Skp trimers may bind the lipase via hydrophobic interactions, but two copies are required for stabilizing the aggregation-prone protein. As the deletion of the *skp* gene in *P. aeruginosa* correlates with the reduced LipA secretion, we suggest that Skp is a crucial factor in the lipase biogenesis pathway.

Folding of the lipase LipA takes place in the periplasm, where the protein is transiently localized and interacts with the specialized chaperone LipH. The chaperone serves for correct positioning of the lid domain of LipA, thus rendering the active enzyme prior its further secretion via T2SS (Rosenau et al., 2004; Pauwels et al., 2006). The non-activated lipase tends to acquire loosely folded intermediate states, which are accessible for proteases and prone to aggregation (Frenken et al., 1993; Rashno et al., 2017). The chaperone-dependent folding is not common among bacterial lipases, and it might be related to the particular structure of the lid domain (Khan et al., 2017). In agreement with the earlier findings of Chiti and co-workers (Rashno et al., 2017, 2018), our data show that the lid domain greatly contributes to LipA aggregation and misfolding into β-stranded structures, thus interfering with the protein biogenesis. As the assembly of the LipA:LipH complex rescues LipA from aggregation to a large extent, two distinct functions can be assigned to LipH, namely stabilization and activation of the client lipase. Speculatively, the chaperone might initially serve to stabilize the lipase in its folded state and hand it over to T2SS, and through the course of the evolution it has gained the propensity to activate the lipase via steric re-positioning of the lid domain.

Several challenges can be envisioned for productive LipA:LipH interactions in the living cell. Firstly, the expression levels of the foldase are substantially lower than those of LipA, so each LipH molecule should carry out multiple chaperoning cycles upon binding nascent lipases, refining the structure and releasing them for further secretion (Rosenau et al., 2004). Secondly, the accessibility of the membrane-anchored foldase for its client may be limited, especially in the crowded periplasmic environment. Finally, the high ionic strength encountered in the sputum of cystic fibrosis patients (Kirchner et al., 2012; Grandjean Lapierre et al., 2017) strongly promotes the aggregation of LipA and may also inhibit the electrostatic interactions at the LipA:LipH interface. Thus, accessory factors, such as non-specific but ubiquitous periplasmic chaperones may be beneficial for biogenesis of the intrinsically unstable lipase. Here, we elucidate that Skp_3_, a conditionally essential protein in *P. aeruginosa* (Lee et al., 2015), is a potent holdase for LipA that efficiently maintains the soluble state of the lipase, blocks the assembly of β-structured aggregates, and ensures secretion of the functional lipase *in vivo*. Notably, even 10-fold excess of Skp_3_ did not inhibit LipA activation and thus allowed LipA:LipH interactions, so we described the chaperone as a factor in LipA biogenesis that steers folding pathways by preventing aggregation events.

Up to date, the vast majority of insights on Skp functioning have been gained from studies on the chaperone from *E. coli*. Differently from other periplasmic chaperones, Skp targets its clients based on the hydrophobicity exposed in the unfolded/misfolded structures rather than a specific sequence motif or conformations of proline residues (Burmann et al., 2013; Schiffrin et al., 2016). The periplasmic concentration of *Ec*Skp may reach hundreds of µM (Tsirigotaki et al., 2017), and the chaperone extensively interacts with unfolded OMPs (Denoncin et al., 2012; Schiffrin et al., 2016; Pan et al., 2020). Although no OMP essentially depends on Skp in the presence of SurA (Denoncin et al., 2012; He & Hiller, 2018), other moonlighting activities have been suggested exclusively for Skp. Recently, the chaperone has been described as an adaptor protein that delivers the misfolded OMP LamB to the periplasmic protease DegP (Combs & Silhavy, 2022). Skp is a potent disaggregase of OmpC and OmpX (Li et al., 2018; Chamachi et al., 2022) and it also demonstrates the holdase and disaggregase activity towards several soluble proteins (Purdy et al., 2007; Wagner et al., 2009; Entzminger et al., 2012). Heterologous expression and secretion of a lipase from *Burkholderia* in *E. coli* could be improved by 36% upon co-expression of Skp, but not SurA (Narayanan et al., 2011). The limited increase in the secretion efficiency may be related to the intrinsically high concentration of Skp in *E. coli* periplasm, while deletion of Skp abolishes LipA secretion, as it is presented in our work. Biochemical analysis suggests that Skp-mediated solubility of the lipase is a decisive factor for the efficient biogenesis of the enzyme.

While the functional insights on Skp of *P. aeruginosa* are sparse (Klein et al., 2019), the chaperone appears to be essential for the survival of bacteria in the sputum media, where the salt concentration exceeds 100 mM (Lee et al., 2015). The chaperone has been detected in multiple proteomics studies (Peng et al., 2005; Rao et al., 2011; Park et al., 2014, 2015; Carruthers et al., 2020), which revealed that the abundance of Skp changes upon switching from the planktonic to the biofilm state, and upon antibiotic treatment. Structural analysis on Skp_3_ of *P. aeruginosa* presented here suggests that its architecture and dynamics resemble those of *Ec*Skp_3_, despite the low sequence similarity and presence of multiple proline residues. *P. aeruginosa* Skp exists as an assembled trimer in solution, and its conformational plasticity and the hydrophobic interior fulfil the requirements for being a promiscuous non-specific holdase chaperone. The SAXS data describe the transient outward motion of the “arm” domains that ensures opening of the interior cavity of the Skp trimer. The aggregation-prone folding intermediates of LipA are likely to be recognized by Skp_3_, kept in the protective hydrophobic cavity and then handed over to LipH for activation.

**FIGURE 9:**
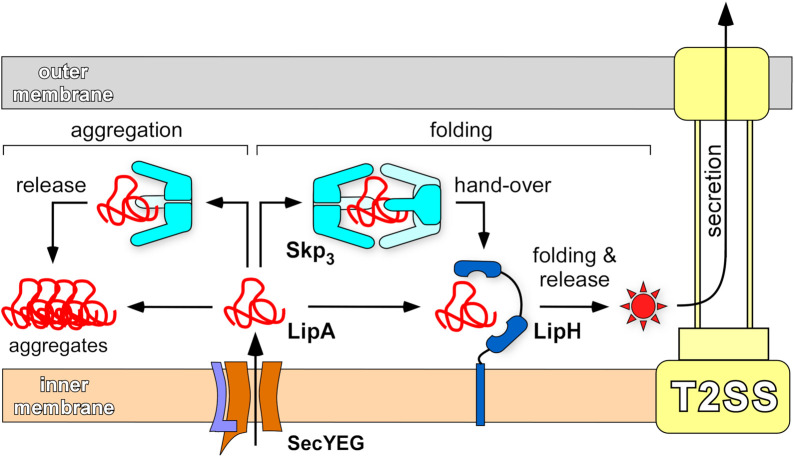
Chaperone-assisted folding of LipA in the periplasm. After the initial translocation *via* SecYEG complex into the periplasm, the aggregation-prone LipA may be directly picked up by the foldase LipH, acquire the native, enzymatically active structure (indicated as a red star) and be transferred to the type II secretion machinery (T2SS). Alternatively, LipA may interact with the abundant trimeric chaperone Skp_3_ that prevents LipA from aggregation, when a complex at 1:2 stoichiometry is formed. Upon its release, LipA may bind LipH and proceed along the folding/secretion pathway. A single copy of Skp_3_ does not ensure the holdase function and does not rescue LipA from aggregation.

The biphasic LipA:Skp_3_ binding with affinities of ∼20 nM and 2 µM is in remarkable agreement with the chaperone interactions with OMPs, where binding of either one or two copies of Skp_3_ has been described (Qu et al., 2007; Schiffrin et al., 2016; Mas et al., 2020; Pan et al., 2020). The close match between the Skp_3_ affinities to different classes of clients is likely due to non-specific hydrophobicity-based interactions. Unexpectedly though, the relatively small protein LipA is able to recruit two trimers of Skp. The exposed hydrophobic areas must be sufficiently large to form the extensive binding interface; however, the relatively low affinity for the second Skp_3_ suggests limited exposure of the lipase beyond the primary LipA.Skp_3_ complex. The high affinity of a single Skp_3_ to LipA is comparable to that of the LipA:LipH complex (Pauwels et al., 2006; Viegas et al., 2020), raising a question about the interplay of these chaperones on the lipase activation pathway. We may speculate that interactions of unfolded LipA with Skp_3_ are associated with rapid capture and release events, while the sterically and electrostatically tuned complex of LipA:LipH is characterized by a low dissociation rate (Viegas et al., 2020). At a large excess of Skp_3_, likely present in the periplasm, a nascent LipA molecule experiences multiple rapid association/dissociation events with monomers and dimers of Skp_3_ before acquiring its specific fold, required for binding to the foldase LipH. The release of the lipase from LipH may be triggered via interactions with components of T2SS. The putative model of LipA interactions and pathways in the periplasm, derived from *in vitro* and *in vivo* analysis, is illustrated by Figure 9.

Our data provide first evidence for interactions of *P. aeruginosa* LipA with the general and highly abundant periplasmic chaperone Skp that result in stabilization of the lipase. It remains to be shown at what stage along the biogenesis pathway LipA requires the involvement of Skp, and whether the chaperone:client interactions are beneficial under particular conditions, such as elevated ionic strengths. Future experiments in the native environment of *P. aeruginosa* combined with *in vitro* analysis will account for the membrane-anchored LipH and its putative competition with Skp for the nascent LipA, as well as the handover of LipA to the T2SS machinery and potential involvement of Skp at that stage.

## Data Availability

The datasets presented in this study can be found in online repositories. The names of the repository/repositories and accession number(s) can be found in the article/Supplementary Material.
