# Supplementary material for: The periplasmic chaperone Skp prevents misfolding of the secretory lipase A from Pseudomonas aeruginosa
Source: Front Mol Biosci. 2022 Oct 24;9:1026724. doi: 10.3389/fmolb.2022.1026724 (PMC9638971; doi:10.3389/fmolb.2022.1026724)
Supplement: Supplementary file 1 [file Table1.DOCX]

**The periplasmic chaperone Skp prevents misfolding
of the secretory lipase A from *Pseudomonas aeruginosa***

Athanasios Papadopoulos ^1^, Max Busch ^1^, Jens Reiners ^2^, Eymen Hachani ^3^,
Miriam Bäumers ^4^, Julia Berger ^5^, Lutz Schmitt ^3^, Karl-Erich Jaeger ^5^, Filip Kovacic ^5^,
Sander H.J. Smits ^2,3^ and Alexej Kedrov ^1, 🖂^

*Supplemental data*

**
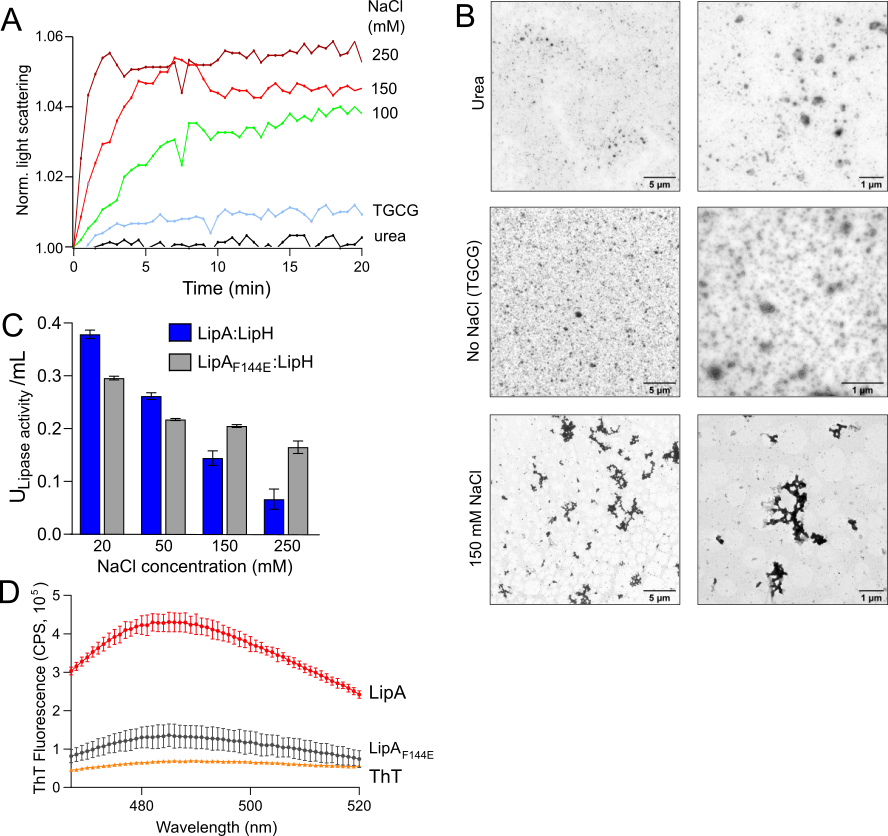
**

**Supplemental Figure 1. Detection of the lipase aggregation.**

**A)** Static light scattering by wild-type LipA at different conditions. In comparison to urea-containing buffer or the low-salt TGCG buffer, light scattering by LipA rapidly increases at the elevated salt concentrations.

**B)** Negative-stain TEM images of the wild-type LipA at different conditions. In comparison to urea-containing buffer or the low-salt TGCG buffer, LipA extensively forms aggregates at the elevated salt concentration (150 mM NaCl). Scale bars are indicated.

**C)** LipH-mediated activity of LipA decreases with the increasing salt concentration, as a consequence of the enhanced lipase aggregation. The activity of the mutant lipase LipA_F144E_ is less affected by the ionic strength.

**D)** LipA aggregation is associated with formation of β-structured aggregates. The intensity of the amyloid/β-structure-sensitive ThT fluorescence (counts per second, CPS) was measured in presence of either the wild-type LipA (red trace) or LipA_F144E_ (black trace) in the TGCG buffer supplemented with 20 mM NaCl. The ThT signal (yellow trace) is shown for comparison. The assay was performed in technical triplicates, the mean values and SD are shown.

**
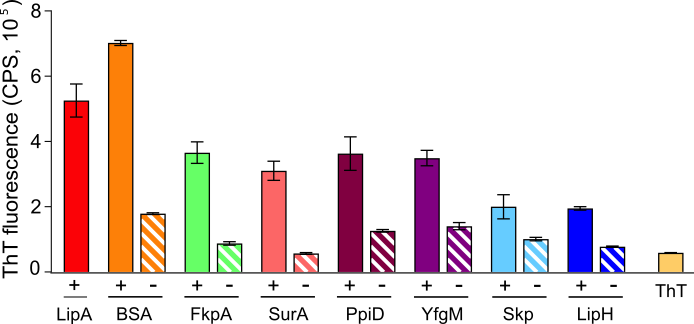
**

**Supplemental Figure 2. ThT fluorescence in the presence of *P. aeruginosa* chaperones**

The intensity of the amyloid/β-structure-sensitive ThT fluorescence was measured for indicated periplasmic chaperones in absence (“-”) and presence (“+”) of the wild-type LipA. BSA was included as a negative control. Signals measured for LipA alone and ThT in absence of proteins are shown. The assays were performed in technical triplicates, the mean values and SD are shown.

**
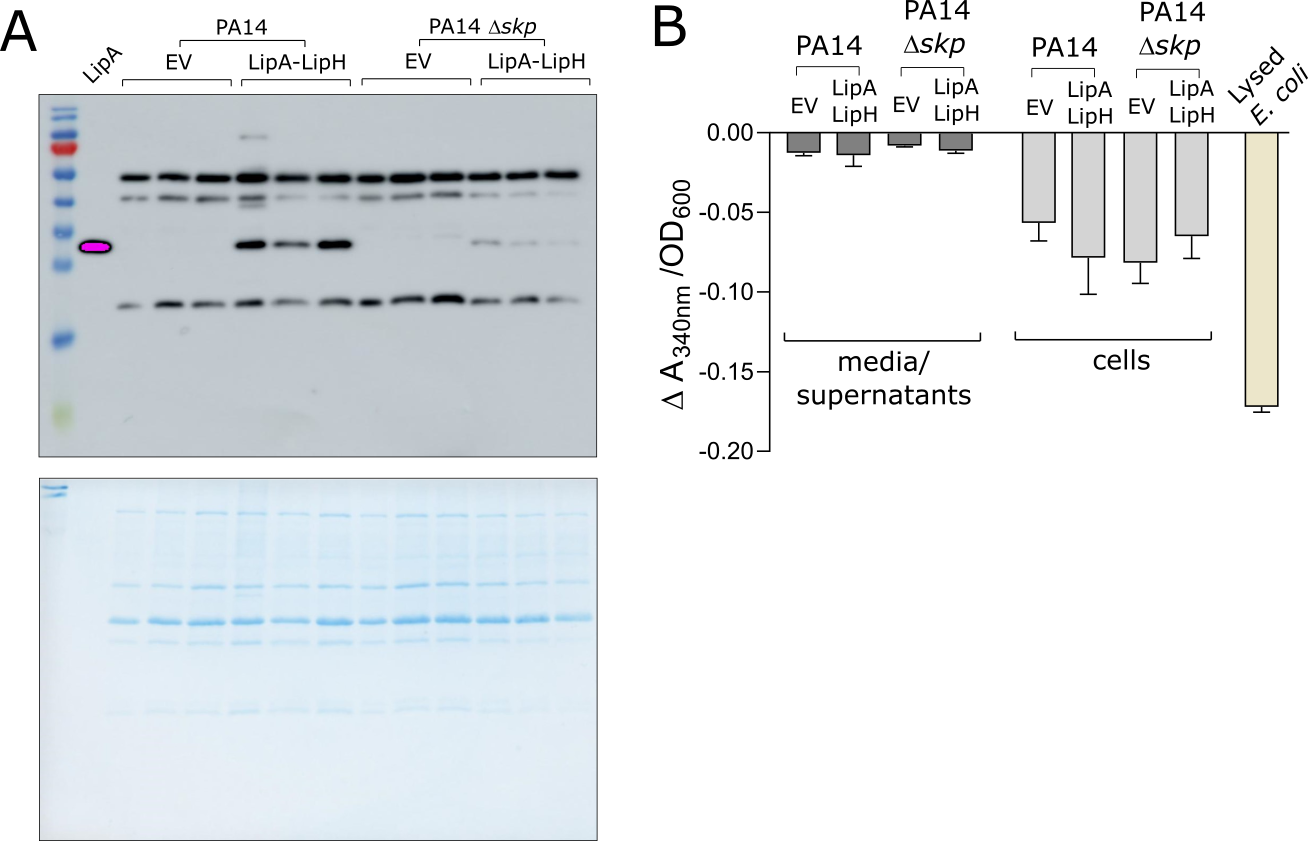
**

**Supplemental Figure 3.**

1. Complete western blot membrane (top) and post-transfer stained SDS-PAGE of *P. aeruginosa* PA14 supernatants.
2. The integrity of *P. aeruginosa* cells was probed via the oxidase activity assay. Conversion of NADH to NAD^+^ was monitored as a decrease in absorbance at 340 nm in the cell-free media and in cells of *P. aeruginosa* PA14 and PA14 Δ*skp* carrying the empty vector (EV, pGUF) and LipA-LipH expression vector, as described. Lysed *E. coli* DH5α cells served as a positive control.

**
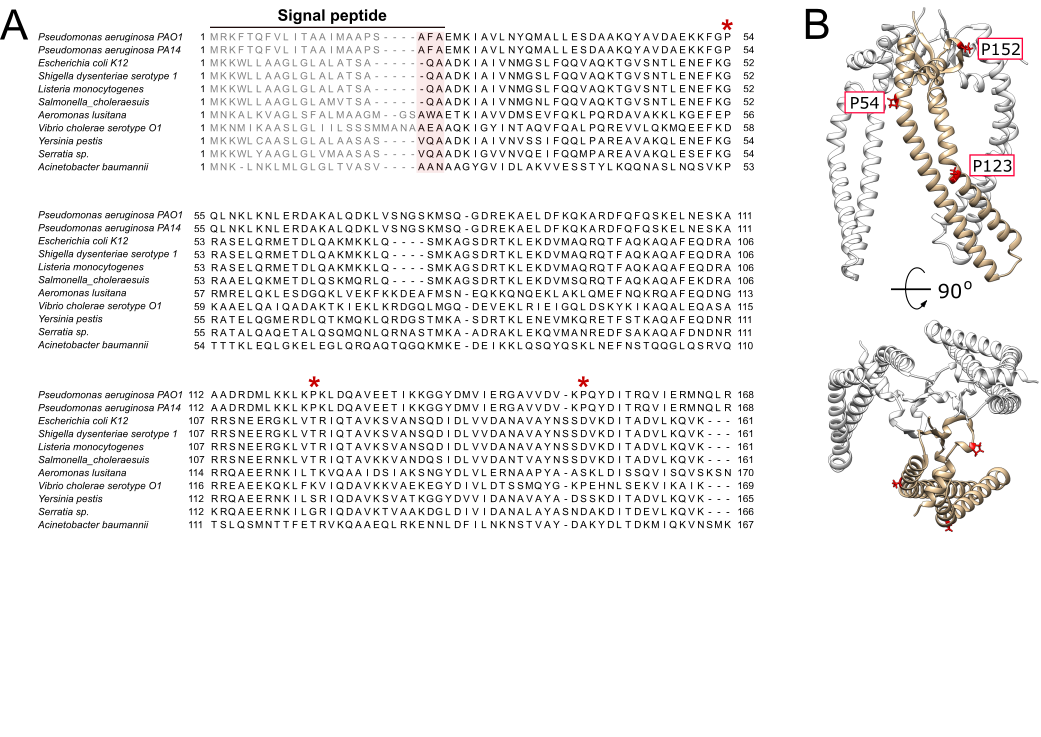
**

**Supplemental Figure 4.**

1. Multiple sequence alignment of Skp chaperones from Gram-negative bacteria. The proline residues within the mature domain of *P. aeruginosa* Skp are indicated. Position of the signal peptide is indicated, as well as the cleavage site for the signal peptidase (pink). The alignment was performed with Clustal Omega utilizing SnapGene Software.
2. Structural model of *P. aeruginosa* Skp_3_ with the proline residues indicated within one subunit (yellow).

**
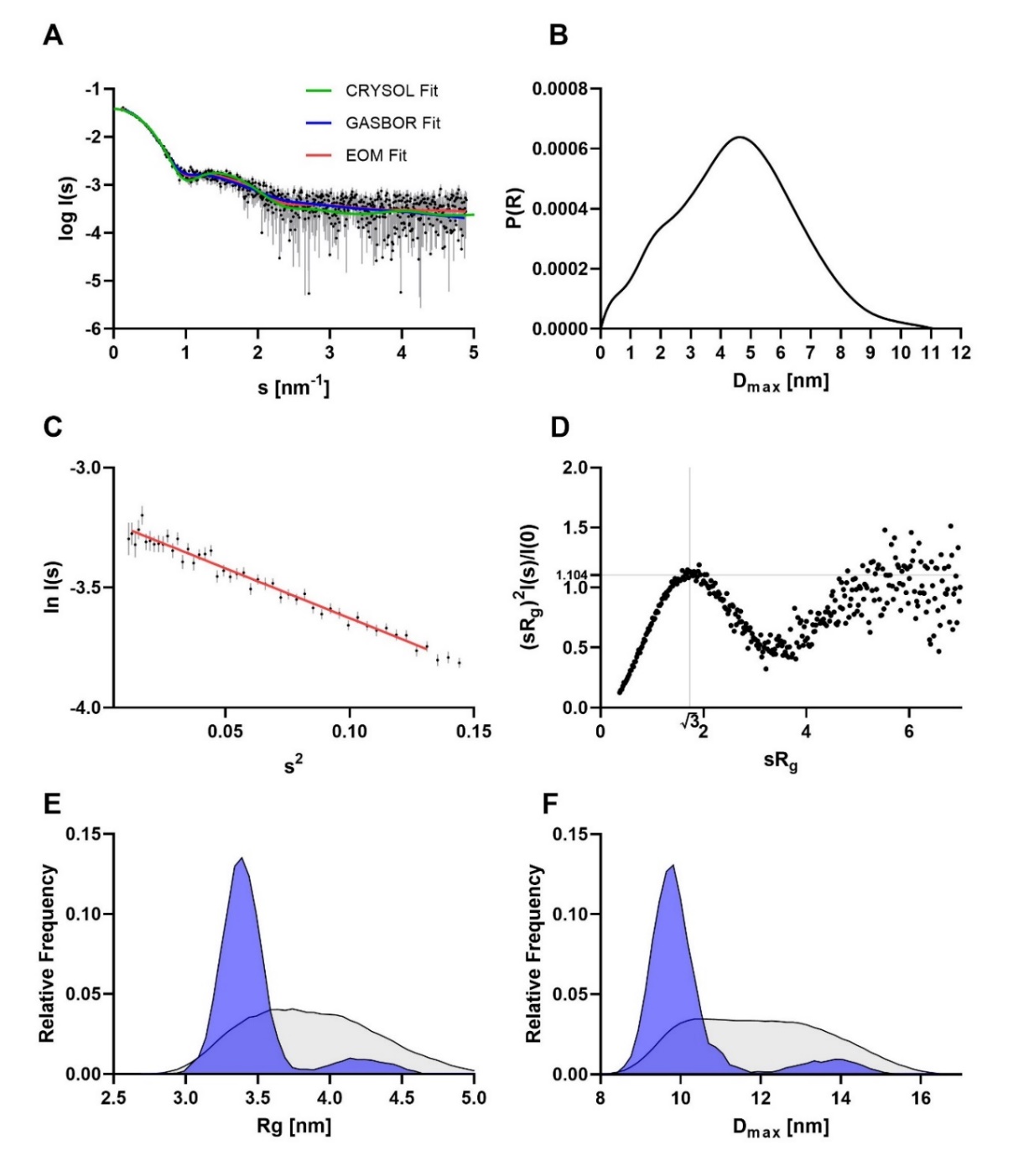
Supplemental Figure 5. Small-angle X-ray scattering data from Skp of *P. aeruginosa*.**

1. Experimental SAXS data curve is shown in black dots with grey error bars. The EOM-based fit is shown as red line (χ^2^ =0.954), the GASBOR fit as blue line (χ^2^ =1.016) and the CRYSOL fit based on AlphaFold 2 model of the Skp trimer as green line (χ^2^ =1.28). The intensity is displayed as a function of momentum transfer s.
2. The corresponding distance distribution function *(p(r)* function).
3. Guinier plot showed a stable Guinier region.
4. Dimension-less Kratky plot of *P. aeruginosa* Skp.
5. *R_g_* distribution calculated by EOM. . RANCH pool is shown in grey and the selected models in blue.
6. *D_max_* distribution calculated by EOM. RANCH pool is shown in grey and the selected models in blue.

**
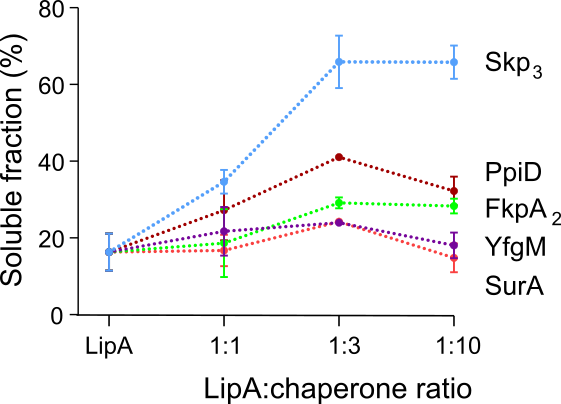
**

**Supplemental Figure 6. Anti-aggregation potential of the periplasmic chaperones determined in LipA sedimentation assay.**

LipA was incubated at 150 mM NaCl and 20 mM Tris-HCl pH 8.0 either alone or with the periplasmic chaperones at the indicated molar ratios, and the fraction of soluble LipA was determined as described.

**Supplemental Table 1. SAXS data summary for *P. aeruginosa* Skp**

| **SAXS Device** | **Xenocs Xeuss 2.0 with Q-Xoom** |
| --- | --- |
| **Data collection parameters** |  |
| Detector | PILATUS 3 R 300K windowless |
| Detector distance (m) | 0.550 |
| Beam size | 0.8 mm x 0.8 mm |
| Wavelength (nm) | 0.154 |
| Sample environment | Low Noise Flow Cell, 1 mm ø |
| *s* range (nm^-1^)^‡^ | 0.05 – 6.0 |
| Exposure time per frame (s) | 600 (24 frames) |
| **Sample** | **Skp** |
| Organism | *Pseudomonas aeruginosa* PAO1 |
| UniProt ID (Range) | Q9HXY5 (23-168) |
| Mode of measurement | batch |
| Temperature (°C) | 15 |
| Protein concentration (mg/ml) | 1.2 |
| Protein buffer | 100 mM NaCl, 10 % glycerol, 20 mM Tris-HCl pH 8.0 |
| **Structural parameters** |  |
| *I*(0) from P(r) | 0.04 |
| *R*_g_ (real-space from P(r)) (nm) | 3.50 |
| *I*(0) from Guinier fit | 0.04 |
| *s-range* for Guinier fit (nm^-1^) | 0.111 – 0.362 |
| *R*_g_ (from Guinier fit) (nm) | 3.53 |
| Points from Guinier fit | 2 - 45 |
| *D*_max_ (nm) | 11.12 |
| POROD volume estimate (nm^3^) | 173.64 |
| **Molecular mass (kDa)** |  |
| From *I*(0) | 55.39 |
| From Qp ^1^ | 54.75 |
| From MoW2 ^2^ | 35.80 |
| From Vc ^3^ | 58.09 |
| Bayesian inference ^4^ | 53.15 |
| From POROD | 86.82 – 108.53 |
| From sequence | 18.37 (monomer)  55.12 (trimer) |
| **Structure Evaluation** |  |
| EOM fit χ^2^ | 0.954 |
| GASBOR fit χ^2^ | 1.016 |
| CRYSOL fit χ^2^ | 1.28 |
| Ambimeter score | 2.199 |
| **Software** |  |
| ATSAS software version ^5^ | 3.0.3 |
| Primary data reduction | PRIMUS ^6^ |
| Data processing | GNOM ^7^ |
| Flexibility analysis | EOM ^8,9^ |
| *Ab initio* modelling | GASBOR ^10^ |
| Structure evaluation | AMBIMETER ^11^ / CRYSOL ^12^ |
| Model visualization | PyMOL ^13^ |
| **SASBDB code** ^14^ | SASDNM5 |

‡s = 4πsin(θ)/λ, 2θ – scattering angle, λ – X-ray wavelength, n.d. not determined

**Supplemental references**

1. Porod, G. Die Röntgenkleinwinkelstreuung von dichtgepackten kolloiden Systemen - I. Teil. *Kolloid-Zeitschrift* **124**, 83–114 (1951).

2. Fischer, H., de Oliveira Neto, M., Napolitano, H. B., Polikarpov, I. & Craievich, A. F. Determination of the molecular weight of proteins in solution from a single small-angle X-ray scattering measurement on a relative scale. *J. Appl. Crystallogr.* **43**, 101–109 (2010).

3. Rambo, R. P. & Tainer, J. A. Accurate assessment of mass, models and resolution by small-angle scattering. *Nature* **496**, 477–481 (2013).

4. Hajizadeh, N. R., Franke, D., Jeffries, C. M. & Svergun, D. I. Consensus Bayesian assessment of protein molecular mass from solution X-ray scattering data. *Sci. Rep.* **8**, 7204 (2018).

5. Manalastas-Cantos, K. *et al.* ATSAS 3.0: expanded functionality and new tools for small-angle scattering data analysis. *J. Appl. Crystallogr.* **54**, 343–355 (2021).

6. Konarev, P. V, Volkov, V. V, Sokolova, A. V, Koch, M. H. J. & Svergun, D. I. PRIMUS: a Windows PC-based system for small-angle scattering data analysis. *J. Appl. Crystallogr.* **36**, 1277–1282 (2003).

7. Svergun, D. I. Determination of the regularization parameter in indirect-transform methods using perceptual criteria. *J. Appl. Crystallogr.* **25**, 495–503 (1992).

8. Bernadó, P., Mylonas, E., Petoukhov, M. V., Blackledge, M. & Svergun, D. I. Structural Characterization of Flexible Proteins Using Small-Angle X-ray Scattering. *J. Am. Chem. Soc.* **129**, 5656–5664 (2007).

9. Tria, G., Mertens, H. D. T., Kachala, M. & Svergun, D. I. Advanced ensemble modelling of flexible macromolecules using X-ray solution scattering. *IUCrJ* **2**, 207–217 (2015).

10. Svergun, D. I., Petoukhov, M. V. & Koch, M. H. J. Determination of domain structure of proteins from x-ray solution scattering. *Biophys. J.* **80**, 2946–2953 (2001).

11. Petoukhov, M. V & Svergun, D. I. Ambiguity assessment of small-angle scattering curves from monodisperse systems. *Acta Crystallogr. Sect. D* **71**, 1051–1058 (2015).

12. Svergun, D., Barberato, C. & Koch, M. H. CRYSOL - A program to evaluate X-ray solution scattering of biological macromolecules from atomic coordinates. *J. Appl. Crystallogr.* **28**, 768–773 (1995).

13. Schrödinger, LLC. *The {PyMOL} Molecular Graphics System, Version~1.8*. (2015).

14. Kikhney, A. G., Borges, C. R., Molodenskiy, D. S., Jeffries, C. M. & Svergun, D. I. SASBDB: Towards an automatically curated and validated repository for biological scattering data. *Protein Sci.* **29**, 66–75 (2020).
